# Genetic dissection reveals distinct contributions of the eS31 N‐terminal domain to translational accuracy in *Saccharomyces cerevisiae*

**DOI:** 10.1111/febs.70510

**Published:** 2026-03-24

**Authors:** Qingxuan Gao, Hiroshi Otsuka, Kei Endo, Koichi Ito

**Affiliations:** ^1^ Department of Computational Biology and Medical Sciences, Graduate School of Frontier Sciences The University of Tokyo Chiba Japan

**Keywords:** ribosomal protein S31 (eS31), ribosome heterogeneity, ribosome stalling, *Saccharomyces cerevisiae*, translational fidelity

## Abstract

The eukaryote‐specific N‐terminal domain of ribosomal protein eS31 is a flexible extension near the decoding center, but its role in translational control has been obscured by pleiotropic defects associated with complete domain deletion. To resolve this ambiguity, we dissected its function at amino acid resolution in *Saccharomyces cerevisiae* using a genetic screen for ribosome stalling at CGA codon repeats. By functionally separating the N‐terminal ubiquitin moiety from the ribosomal domain, our screen bypassed artifacts and identified a critical cluster of basic residues (K79–K82) within this region. Charge‐reversing point mutations in this cluster alleviate stalling not by impairing ribosome‐associated quality control, but by reducing decoding fidelity, as supported by paromomycin sensitivity and genetic suppression by the loss of the eEF2 diphthamide modification. Crucially, the mutant lacking the entire domain operates through a fundamentally distinct mechanism, exhibiting a severe defect in termination fidelity and a paradoxical requirement for wild‐type eS31 that suggests a global structural perturbation. Our findings thus resolve the function of the N‐terminal domain of eS31 into two distinct modes: a residue‐specific, electrostatic role in fine‐tuning elongation fidelity, and a broader structural role for the entire domain in maintaining ribosome integrity for accurate termination.

## Graphical abstract

The eukaryote‐specific N‐terminal domain (NTD) of eS31 uses two distinct strategies to maintain translation fidelity. During elongation, a positively charged “hotspot” fine‐tunes the selection of incoming aa‐tRNA. During termination, the entire NTD acts as a structural scaffold to ensure the correct positioning of the release factor eRF1. Our genetic dissection reveals how a single ribosomal protein element can enforce fidelity at two different stages of translation.

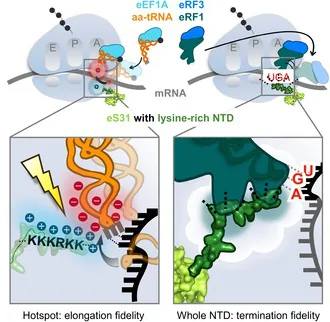

**Abbreviations**
eS31: eukaryotic ribosomal protein S31
NTD: N‐terminal domain
RD: Ribosomal Domain
ΔNTD: N‐terminal domain deletion
RQC: Ribosome‐associated Quality Control
NGD: No‐Go Decay
NSD: Non‐Stop Decay
OE: overexpression
WT: wild‐type

## Introduction

Ribosomes are universally conserved ribonucleoprotein complexes that decode mRNAs into proteins. While the catalytic core of the ribosome is shared across all domains of life, eukaryotic ribosomes have evolved into complex regulatory platforms, acquiring numerous eukaryote‐specific proteins and extensions that are concentrated on the ribosomal surface [1, 2, 3, 4]. These additional components enhance the ribosome's regulatory capacity. For instance, the eukaryote‐specific ribosomal protein RACK1 (Asc1 in yeast) functions as a scaffold on the 40S subunit, recruiting auxiliary factors such as signaling kinases and E3 ubiquitin ligases, which are crucial for coordinating translation with cellular quality control pathways [5, 6, 7]. Notably, many of these additions manifest as unstructured N‐ and C‐terminal extensions that are highly mobile and thus ideally positioned to mediate dynamic interactions with various translation factors and regulatory proteins [8, 9, 10]. A prime example is ribosomal protein eS31 (also called Rps31 in yeast, RPS27a in mammals), a component of the 40S subunit [11]. Beyond its canonical role in ribosome biogenesis, eS31 has crucial extra‐ribosomal functions; for instance, in higher eukaryotes, free eS31 is known to regulate the MDM2‐p53 tumor suppressor axis and is implicated in numerous cancers and viral responses [12, 13, 14, 15]. While these functions highlight the biomedical significance of eS31, the fundamental role of its ubiquitin‐fusion nature and the basic N‐terminal tail in ribosomal function is evolutionarily conserved in *S. cerevisiae*, making it the primary model organism for elucidating these mechanisms. This broad repertoire of functions underscores the need to understand the molecular mechanisms governing eS31's activity with high resolution. Indeed, even within the ribosome, eS31 is implicated as a dynamic component in stress responses; for instance, its C‐terminal domain is a target for ubiquitination under oxidative stress and is involved in a quality control network that degrades stalled translation factors [16, 17].

The synthesis of eS31 as a fusion protein with an N‐terminal ubiquitin moiety is a highly conserved feature across eukaryotes [18]. In yeast, eS31 is encoded by the *UBI3* gene as such a precursor protein [19]. Following its co‐translational cleavage, eS31 is incorporated into the ribosome [11, 20], where its conserved C‐terminal domain adopts a compact globular fold accompanied by a flexible N‐terminal extension (eS31 G77‐K99) unique to eukaryotes [4, 18]. eS31 is localized to the beak domain of the 40S ribosomal subunit, which in eukaryotes is composed of helix h33 of the 18S rRNA and the ribosomal proteins eS10, eS12, and eS31, and is positioned on the solvent‐exposed surface of the 40S head domain [21]. This region has been implicated in interactions with regulatory factors such as Gcn1 during translation stress responses [22]. The N‐terminal domain (NTD) of eS31 extends from the 40S beak domain with its tip dipping into the mRNA channel (Fig. 1A). This strategic placement also situates the NTD near other key translation factors such as eEF2, eRF1, and Dom34 [23, 24, 25, 26] (Fig. 1B). However, elucidating the specific functions of the NTD has been challenging. Previous studies that relied on deleting the entire domain (eS31ΔNTD) were confounded by pleiotropic defects, including impaired ribosome biogenesis due to the loss of a nuclear localization signal [18]. Consequently, it remains unresolved whether the known fidelity defects of eS31ΔNTD mutants are a direct consequence of losing a specific regulatory surface or a secondary effect of a perturbed ribosome structure. A high‐resolution genetic approach is therefore needed to deconstruct the functions encoded within the eS31 NTD.

**Fig. 1:**
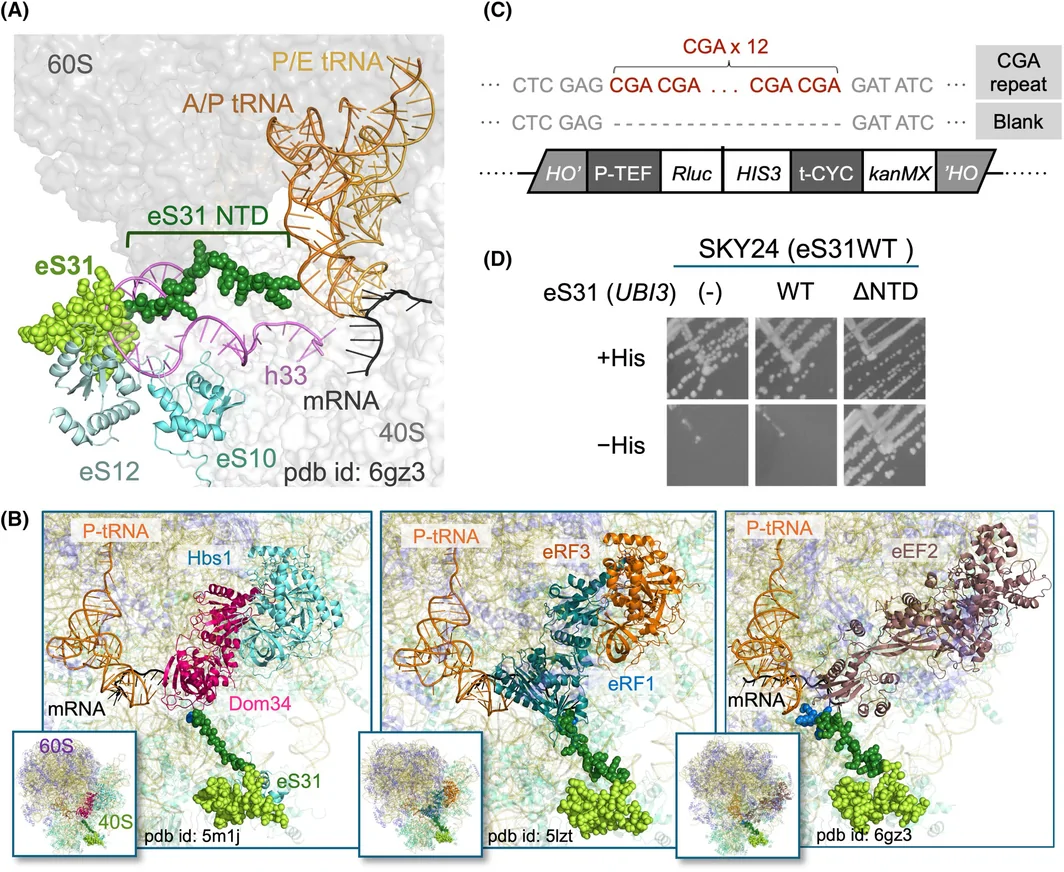
The role of the eS31 N‐terminal domain in CGA‐repeat‐mediated translational stalling. (A) Structure of ribosomal protein eS31 within the eukaryotic 40S subunit beak domain (pdb id: 6gz3) [23]. The C‐terminal globular domain (light green) and the eukaryote‐specific N‐terminal domain (NTD, dark green) are highlighted. (B) Structural context of the eS31 N‐terminal domain. Representative cryo‐EM structures illustrating the orientation of the eS31 N‐terminal domain (dark green) relative to key translation factors bound at or near the ribosomal A‐site. In all panels, the ribosome is shown as a transparent cartoon, with ribosomal RNA colored yellow, large subunit proteins slate, and small subunit proteins greencyan. The strategic proximity of the eS31 NTD to these factors, including Dom34–Hbs1 (pdb id: 5m1j) [26], eRF1 (pdb id: 5lzt) [24], and eEF2 (pdb id: 6gz3) [23], highlights its potential role in translational control. (C) Schematic of the CGA_12_–*HIS3* reporter used to screen for stalling‐bypass phenotypes. An array of 12 consecutive CGA codons, a potent stalling sequence, is inserted upstream of the *HIS3* coding region. In wild‐type cells, ribosomes stall on the CGA repeats, preventing expression of the downstream His3 protein and thus conferring a His^−^ phenotype. Conversely, mutations that allow ribosomes to bypass this stall result in HIS3 synthesis and scorable growth on medium lacking histidine. The reporter construct was integrated at the *HO* locus as previously described [27]. (D) Growth assay of the CGA_12_–*HIS3* reporter strain expressing an empty vector, wild‐type eS31 (WT), or an N‐terminal deletion mutant (eS31ΔNTD). Images represent cropped sections from standard 12 cm agar plates and are representative of results obtained from at least three independent transformants tested in parallel, all of which exhibited reproducible growth phenotypes. Full uncropped plate images are available in Fig. S1.

Eukaryotic cells have evolved surveillance pathways to resolve prolonged ribosome stalling, which can be triggered by various obstacles such as strong mRNA secondary structures, rare codon clusters, or limited tRNA availability [28, 29, 30, 31, 32]. We then employed a high‐sensitivity genetic reporter in *Saccharomyces cerevisiae* that leverages the severe translational stalling induced by CGA codon repeats [33, 34, 35, 36, 37, 38]. This stalling arises from a direct structural impediment within the ribosomal A‐site, making the system an exquisitely sensitive biosensor for perturbations in decoding fidelity. Previous studies have shown that full‐length CGA‐stalling reporters persist even in wild‐type cells, suggesting that a subset of stalled ribosomes evade canonical degradation [38, 39]. This stalling is exacerbated by both the inefficient decoding by its cognate tRNA, whose availability and modification are limited [40], thereby allowing premature termination events triggered by misrecognition of CGA codons by the release factor eRF1 to compete with the canonical decoding pathway [41, 42]. Persistent stalling triggers ribosome‐associated quality control (RQC), but defects in this process allow bypass of the CGA tract and expression of a downstream *HIS3* reporter, conferring a screenable growth phenotype. To specifically isolate the ribosomal functions of eS31 and bypass confounding artifacts from its ubiquitin‐fusion precursor (*UBI3* gene), we implemented a parallel screening strategy, performing random mutagenesis on both the full‐length protein and on a construct expressing only the eS31 ribosomal domain (RD). This approach enabled us to pinpoint critical residues at an amino acid resolution. Using this high‐resolution approach, we demonstrate that a specific cluster of basic residues (K79–K82) within the NTD fine‐tunes decoding fidelity through an electrostatic mechanism, distinct from canonical RQC pathways. Crucially, we reveal that the complete deletion of the NTD (eS31 ΔNTD) operates through a fundamentally different mechanism, causing a severe defect in termination fidelity and inducing a global translational disruption that paradoxically requires the presence of wild‐type eS31. Our findings thus dissect the eS31 NTD's function into two distinct modes: a residue‐specific role in modulating elongation fidelity and a broader structural role in maintaining ribosome integrity for accurate termination.

## Results

### A genetic screen identifies a key residue cluster in the eS31 NTD that alleviates CGA‐induced stalling

To investigate the role of the eS31 NTD in translational control, we first tested a construct lacking the entire domain (eS31ΔNTD) in our CGA_12_–*HIS3* reporter strain (hereafter referred to as SKY24) (Fig. 1C, Table S1). This mutant conferred robust growth on histidine‐deficient medium, indicating a bypass of CGA‐induced stalling and implicating the NTD in modulating ribosome progression on problematic codons (Fig. 1D). However, this result is difficult to interpret due to the known pleiotropic effects of whole‐domain deletions, specifically the impairment of ribosome biogenesis and nuclear import of 40S precursors [18, 20], which can obscure direct translational functions. To isolate novel mutations that cause a similar stalling bypass phenotype, we employed a dominant‐negative genetic screen. This strategy is based on the principle that ribosomal proteins expressed from a plasmid can be incorporated into the ribosome pool, competing with their wild‐type counterparts expressed from the chromosome. If a mutant protein impairs a specific ribosomal function—in this case, the ability to stall efficiently at CGA repeats—it will confer a dominant, selectable phenotype (i.e., growth on medium lacking histidine) even in the presence of the wild‐type protein. This approach allows for the identification of functionally critical residues without the immediate need to create a gene knockout, which can sometimes be lethal or cause complex secondary defects. The candidates isolated from this dominant‐negative screen were subsequently validated in a *UBI3* knockout background to confirm their intrinsic functions as the sole source of eS31.

To identify the key residues at higher resolution, we first performed error‐prone PCR mutagenesis (see Materials and Methods for detail) on the full‐length *UBI3* coding sequence (see Fig. 2A for schematics of the eS31 constructs used throughout this study). This screen yielded 33 independent dominant‐negative candidates, but strikingly, all of them mapped to the ubiquitin domain (Fig. 2B, Table 1A). In this pilot‐scale screen, the substitutions exclusively mapped to three residues within the ubiquitin domain: I36, K63, and G76. These mutations are known to affect critical functions, including the E1/E2 enzyme interface (I36) and the ubiquitin cleavage site (G76), and are consistent with disrupting ubiquitin‐dependent pathways [27]. While this validated our screen's sensitivity, the predominance of these hits obscured the ribosomal functions of eS31.

**Fig. 2:**
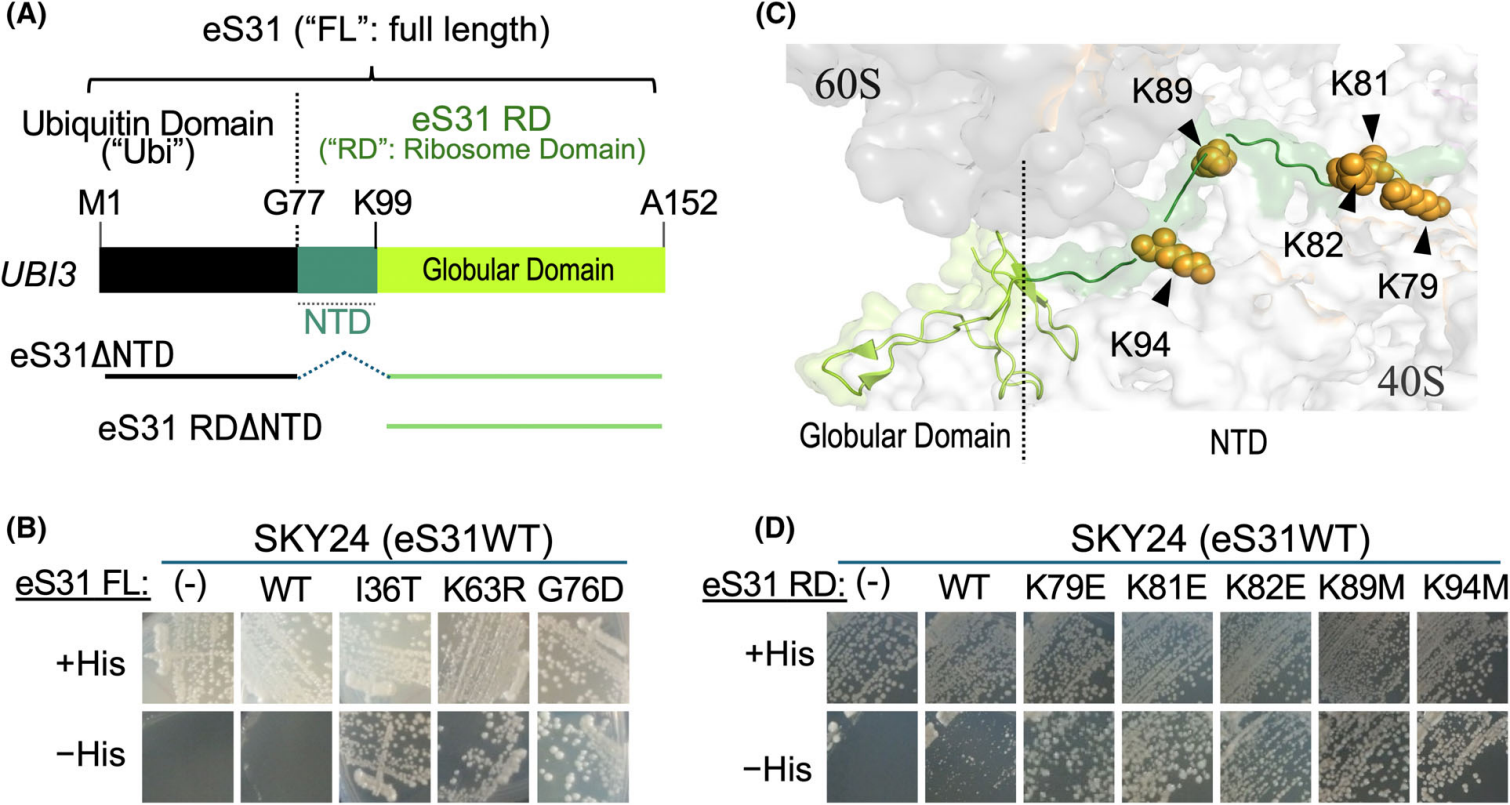
Growth phenotypes of identified eS31 mutants in the CGA_12_–*HIS3* reporter strain. (A) Schematic of *UBI3* showing the ubiquitin domain (M1–G76, black) and the ribosomal domain (G77–A152, light green). The full‐length construct (including ubiquitin) is denoted “eS31,” whereas the ribosome‐incorporated portion is denoted “eS31 RD”. (B) Growth of the CGA_12_–*HIS3* reporter strain transformed with empty vector or plasmids expressing identified eS31 ubiquitin domain mutants. (C) Structural mapping of residues identified in the ribosomal domain, highlighted within the eS31 NTD on the 40S subunit (pdb id: 6gz3) [23]. (D) Growth of the CGA_12_–*HIS3* reporter strain transformed with empty vector or plasmids expressing identified eS31 ribosomal‐domain mutants. For the growth assays shown in (B) and (D), images represent cropped sections from standard 12 cm agar plates and are representative of results obtained from at least three independent transformants tested in parallel, all of which exhibited reproducible growth phenotypes. Full uncropped plate images are available in Fig. S1.

**Table 1:** eS31 mutations.

Mutation	No. of isolates
(A) Mutations by error‐prone PCR on full‐length eS31	
I36T	28
K63Q	1
K63R	1
K63T	1
G76D	2
(B) Mutations by error‐prone PCR on eS31 RD	
K79E	5
K79E/K81E	4
K81E	4
K82E	2
K89M	2
K94M	4

To circumvent this bias, we devised a parallel screening strategy using a construct that expresses only the eS31 ribosomal domain (eS31 RD), which begins at G77. This RD‐specific screen was highly successful, yielding a distinct set of mutations that all clustered within the flexible NTD at positions K79, K81, K82, K89, and K94 (Table 1B; Fig. 2C,D). Notably, all mutations isolated in this screen occurred at lysine residues, prompting the question of whether this effect is specific to these particular lysines or involves other residues within the NTD. This result pinpointed a specific region of the NTD as a regulatory hub for ribosome progression on CGA repeats, prompting us to focus our subsequent analysis on these newly identified mutations.

### Systematic mutagenesis defines a functional hotspot in the eS31 NTD

Our initial screen pinpointed several lysine residues as crucial for ribosome progression on CGA repeats. To systematically map the functional landscape of the entire NTD at higher resolution and determine the specificity of this effect, we first substituted every lysine from residue 78 to 99 with glutamic acid (K → E) in eS31 RD construct. This comprehensive analysis revealed that a strong stalling‐bypass phenotype was tightly localized to residues K79, K81, and K82, with weaker effects at the boundary residues K78 and K83 (Fig. 3A). Residues from K89 onwards were inactive. Notably, we observed that the wild‐type eS31 RD construct conferred a slightly higher background growth than an empty vector, an effect not seen with full‐length wild‐type eS31. This is likely a minor pleiotropic effect resulting from bypassing the native ubiquitin processing pathway.

**Fig. 3:**
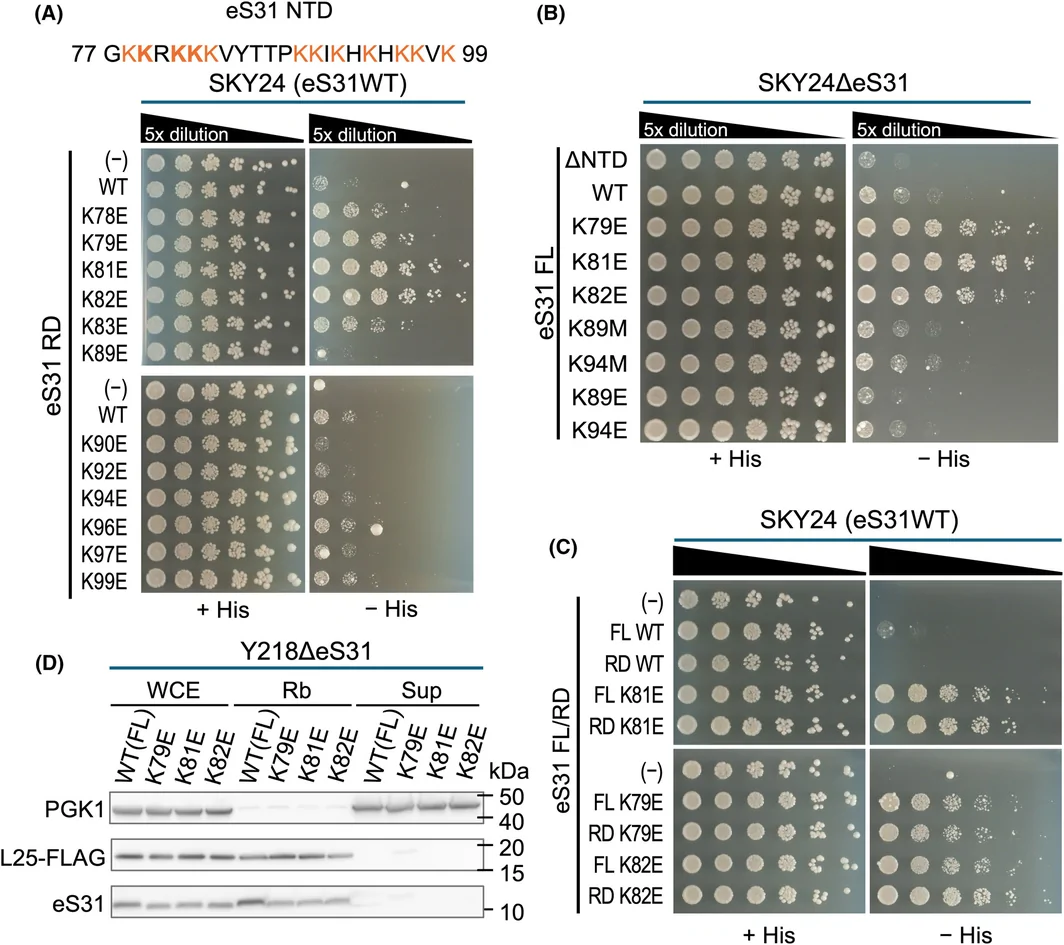
Point mutations in the eS31 NTD bypass stalling without affecting ribosome biogenesis. (A) Systematic functional mapping of the eS31 NTD. Plasmids expressing eS31 RD variants with systematic lysine‐to‐glutamic acid (K → E) substitutions were assayed in the CGA_12_–*HIS3* reporter strain. A strong growth phenotype is confined to residues K79, K81, and K82. (B) eS31 NTD point mutants support cell viability. A *UBI3* knockout strain carrying a URA3‐marked plasmid expressing wild‐type eS31 was transformed with TRP1‐marked plasmids expressing the indicated eS31 mutants. The URA3‐marked plasmid was then lost by plasmid shuffling using 5‐fluoroorotic acid (See Methods). Yeast strains expressing the indicated eS31 variants as the sole source of eS31were serially diluted 5‐fold and spotted onto SC plates lacking tryptophan (to select for the TRP1‐marked plasmids). Plates were prepared with (+His) or without (−His) histidine as indicated in (A), (B) and (C). (C) The stalling‐bypass phenotype of the key point mutants is consistent between the full‐length (FL) and ribosomal domain (RD)‐only constructs. Growth assay comparing the indicated mutants expressed from either FL or RD plasmids. (D) Western blot analysis of eS31 expression and ribosome incorporation in Y218ΔeS31 strain (Table S1). Whole‐cell extracts (WCE), ribosome pellets (Rb; obtained by sucrose cushion centrifugation), and post‐centrifugation supernatants (Sup) were analyzed. PGK1 served as a cytoplasmic control, and L25‐FLAG as a ribosomal marker. Details see Methods. Growth assays in panels (A–C) are representative of at least three independent biological replicates using independently generated transformants. Western blotting in panel (D) was repeated at least three times with independent biological samples, yielding consistent results.

Having identified this key cluster, we then sought to validate the candidates from our original RD‐specific screen in the more physiologically relevant context of the full‐length *UBI3* construct. We reconstructed all five mutations (K79E, K81E, K82E, K89M, K94M) and evaluated their viability as the sole source of eS31 in a *UBI3* knockout strain (Fig. 3B, hereafter referred to as SKY24ΔeS31). First, we confirmed that the charge‐reversing mutants (K79E, K81E, and K82E) could support normal cell growth as the sole source of eS31 in SKY24ΔeS31 (Fig. 3B). Consistent with our systematic mapping, these mutants also retained their strong stalling‐bypass phenotype when expressed in the SKY24 wild‐type background (Fig. 3C). In contrast, the K89M and K94M mutations lost their stalling‐bypass phenotype in this context. These results definitively indicate that the functional hotspot for fine‐tuning elongation fidelity is strictly delimited to the K79–K82 cluster, whereas residues from K89 onwards do not contribute to this specific regulatory mode.

Finally, to ensure that the observed phenotypes of these key mutants were not due to altered protein expression or stability, we performed western blot analysis. This showed that the K79E, K81E, and K82E proteins were expressed and incorporated into 40S subunits at wild‐type levels (Fig. 3D). These results strongly indicate that the mutations specifically affect the translational function of eS31 without causing global defects in protein stability or ribosome biogenesis. Furthermore, the robust growth of these mutants in a genetic background where eS31 is essential (Figs 3B, 7A; Fig. S2), combined with their wild‐type levels of incorporation into ribosome fractions (Fig. 3D), provides compelling evidence that the mutant proteins are successfully imported and assembled into functional ribosomes. Although the K79–K82 cluster has been suggested to overlap with the NLS, our data align with previous findings that point mutations in this region do not elicit the severe biogenesis defects characteristic of complete domain deletion [18].

Collectively, these analyses allowed us to conclusively define a functional hotspot within the eS31 NTD, composed of a key cluster of basic residues spanning K79 to K82.

### The positive charge of the K79–K82 cluster governs ribosome stalling

Our systematic mutagenesis identified the K79–K82 cluster as a functional hotspot. To quantify the magnitude of this effect and understand the interplay between these residues, we employed a dual‐luciferase reporter with an intervening CGA_12_ tract (Fig. 4A, top). Consistent with the growth assays, the K81E and K82E single mutants significantly increased the translation of the downstream reporter, confirming an enhanced bypass of the stalling sequence (Fig. 4A, middle panel). We next constructed double mutants to investigate potential additive or synergistic effects, noting that the K79E/K81E variant was among those isolated in our initial screen. However, the phenotypes of the double mutants were not stronger than that of the most potent single mutant, K81E, suggesting that although multiple residues contribute to the integrity of this functional hub, a single charge inversion is sufficient to elicit a maximal bypass effect (Fig. 4A, bottom panel). Intriguingly, the K79E/K82E combination resulted in a weaker phenotype than the K82E single mutant, implying a potential negative structural interaction.

**Fig. 4:**
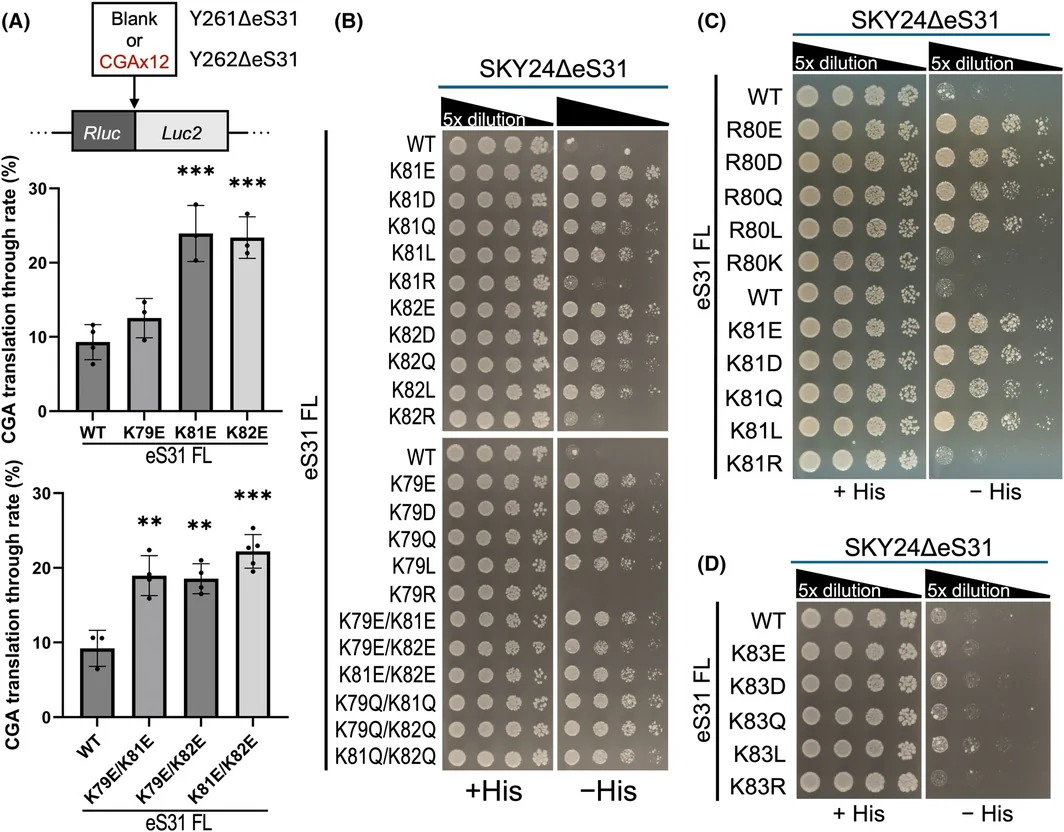
The K79–K82 cluster functions as a cooperative unit to modulate translation. (A) Quantification of CGA‐repeat bypass by dual‐luciferase reporter assay. Strains expressing the indicated eS31 NTD single mutants (top) or double mutants (bottom) were analyzed. The CGA translation through‐rate was calculated as the ratio of Luc2/Rluc from the CGA_12_ reporter (strain Y262ΔeS31), normalized to the ratio from a blank control construct lacking the CGA repeats (strain Y261ΔeS31). Data are shown as mean ± SD. Each dot represents an independent biological replicate (*n* = 3–5 per condition, as indicated). Statistical significance was assessed by one‐way ANOVA with Dunnett's post‐test compared to WT (**P < 0.01, ***P < 0.001). (B) The electrostatic property of the NTD hotspot is critical for its function. Growth assay of the SKY24ΔeS31 strain (a *UBI3* knockout derivative of SKY24), in which the indicated eS31 FL variants were expressed as the sole source of eS31. In the top panel, key lysine residues (K81, K82) were substituted with amino acids of varying physicochemical properties: arginine (R; positive), aspartate (D; negative), glutamine (Q; uncharged polar), or leucine (L; hydrophobic). In the bottom panel, the effects of double mutations are shown. A bypass phenotype is primarily observed when the positive charge is removed or reversed, whereas preserving the charge (K → R) has no effect. Yeast strains expressing the indicated eS31 variants were serially diluted 5‐fold and spotted onto SC plates. The first spot corresponds to the 10^−1^ dilution of the normalized culture (OD_600_ = 0.1) in (B–D). (C) The stalling‐bypass phenotype is charge‐dependent and not specific to lysine. A *UBI3* knockout CGA_12_‐*HIS3* reporter strain expressing eS31 R80 variants was compared to corresponding K81 variants. Charge reversal at R80 (R80E/D) confers a strong growth phenotype, whereas charge preservation (R80K) does not. (D) The phenotype is highly localized. Under the same conditions as in (C), substitutions at the adjacent K83 residue had no significant effect on growth, demonstrating the specificity of the K79–K82 functional hotspot. Growth assays shown in panels (B–D) are representative of at least three independent biological replicates using independent transformants, which yielded comparable results.

Having confirmed the stalling‐bypass effect quantitatively, we next investigated the underlying physicochemical properties responsible for this phenotype. Our screen implicated the positive charge of the K79–K82 hotspot as critical for its function. To test this hypothesis directly, we systematically mutated key residues to amino acids with varying properties. The results were unambiguous: the stalling‐bypass phenotype was driven entirely by the local electrostatic charge. Substitutions that reversed (K → D/E) or neutralized (K → Q) the positive charge consistently produced a strong phenotype, whereas substitutions that preserved the charge (K → R) had no effect (Fig. 4B). Consistent with this charge‐dependent mechanism, mutating the other basic residue in this cluster, R80, also conferred a strong phenotype (Fig. 4C). Furthermore, this charge‐dependent effect was highly localized, as mutations at the adjacent K83 residue had a minimal impact (Fig. 4D).

Collectively, this systematic analysis demonstrates that the stalling‐bypass phenotype is driven by the specific electrostatic properties of this functional hotspot.

### eS31 NTD mutations act upstream of canonical ribosome quality control pathways

The stalling‐bypass phenotype conferred by eS31 NTD mutations is reminiscent of defects in ribosome‐associated quality control (RQC) factors. For instance, mutations in the ribosomal protein S20 or the E3 ligase Hel2 similarly restore *HIS3* expression from our CGA_12_ reporter by impairing ribosome rescue pathways [27, 43]. This prompted us to investigate whether the mutations alleviate stalling by directly impairing the canonical RQC machinery. First, we tested for general ribosome rescue defects using a non‐stop decay (NSD) reporter strain (YWH‐1) (Fig. 5A). NSD is triggered by ribosomes reaching the 3′ end of an mRNA lacking a stop codon, a process that shares core rescue factors with the no‐go decay (NGD) pathway [44, 45, 46, 47, 48, 49]. Unlike the *ski7Δ* positive control, the eS31 mutants failed to restore growth, indicating that they do not cause a general disruption of the NSD pathway (Fig. 5B).

**Fig. 5:**
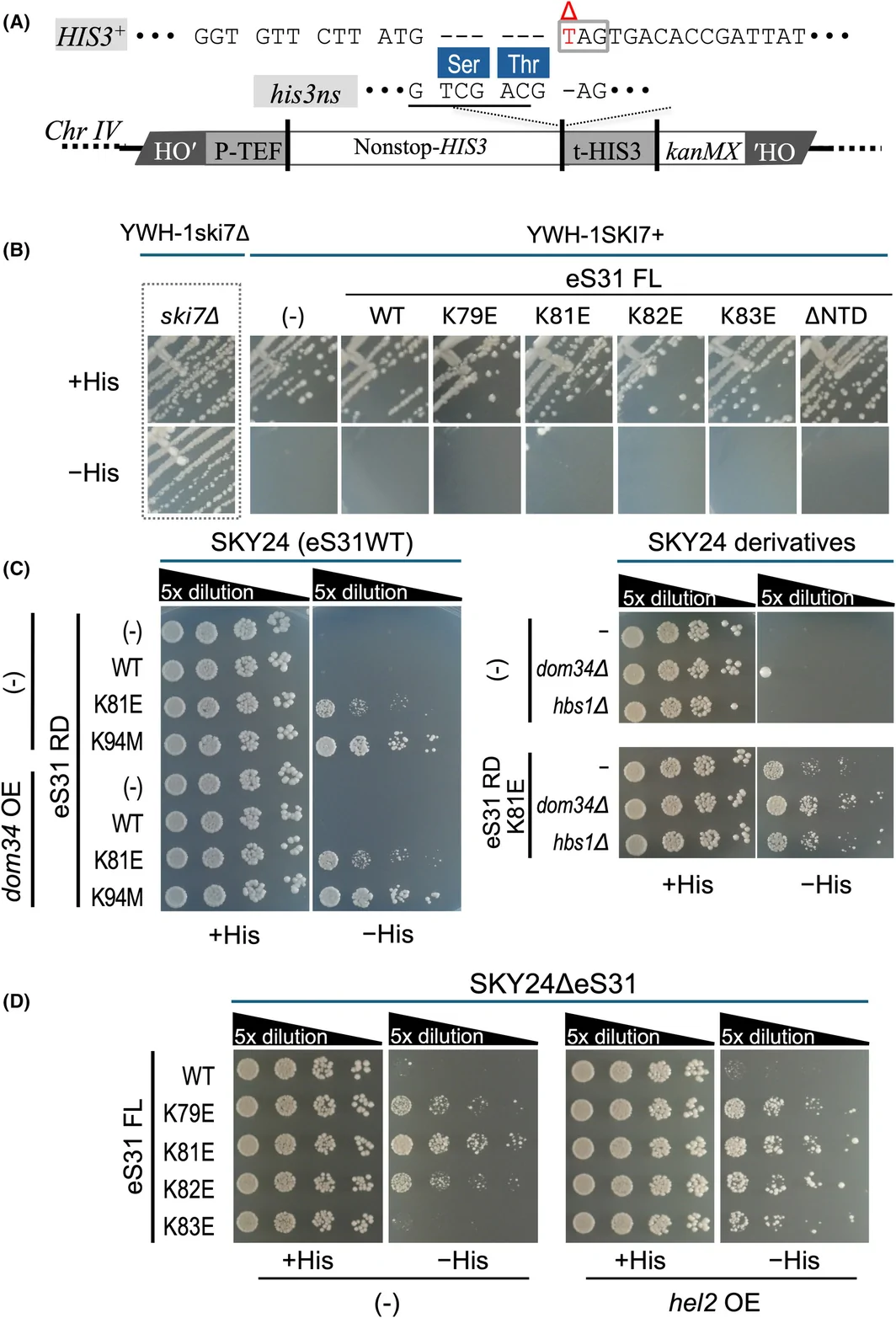
eS31 NTD mutations bypass stalling without disrupting canonical ribosome quality control pathways. (A) Schematic of the non‐stop *HIS3* (*his3ns*) reporter used to assay Non‐Stop Decay (NSD) function. In this system, a single‐base deletion in the stop codon generates a non‐stop transcript. In wild‐type cells, this transcript is recognized and degraded by the NSD pathway, thereby preventing translation of the *HIS3* coding sequence and conferring a His^−^ phenotype. Conversely, genetic defects in the NSD pathway allow for aberrant translation of the non‐stop transcript, resulting in HIS3 expression and a scorable growth phenotype on medium lacking histidine [45, 48]. (B) eS31 NTD mutants do not impair NSD. Growth assay of a *his3ns* reporter strain expressing the indicated eS31 variants. Growth on –His medium is not restored, unlike in the ski7Δ positive control (dashed box), indicating that the NSD pathway remains intact. The strain background is SKI7^+^ unless otherwise noted. Images represent cropped sections from standard 12 cm agar plates. Full uncropped plate images are available in Fig. S1. (C) eS31 NTD mutants do not genetically interact with key No‐Go Decay (NGD) factors Dom34 and Hbs1. Growth of CGA_12_–*HIS3* reporter strains was assessed with plasmid‐based overexpression (OE) or deletion of *DOM34* and *HBS1*. The left panel shows strains co‐transformed with a URA3‐marked Dom34 OE plasmid or an empty vector. The right panel shows SKY24 strains with the indicated gene deletions. (D) Hel2 overexpression does not suppress the eS31 mutant phenotype. *UBI3* knockout CGA_12_–*HIS3* reporter strains expressing the indicated eS31 variants were co‐transformed with an empty vector or a URA3‐marked Hel2 overexpression (OE) plasmid. Hel2 overexpression fails to suppress the growth of the eS31 mutants on –His medium. Yeast strains expressing the indicated eS31 variants were serially diluted 5‐fold and spotted onto SC plates. The first spot corresponds to the 10^−1^ dilution of the normalized culture (OD_600_ = 0.1) in (C) and (D). Growth assays shown in panels (B–D) are representative of at least three independent biological replicates using independent transformants, which yielded comparable results.

We next focused specifically on the NGD pathway, which is directly activated by CGA‐induced stalling. We systematically tested for genetic interactions with key NGD factors but detected no significant interactions. Neither deletion nor overexpression of the ribosome‐dissociating factors Dom34–Hbs1 altered the eS31 mutant phenotype (Fig. 5C). Furthermore, the phenotype was not suppressed by overexpression of the E3 ligase Hel2; if anything, it was slightly enhanced (Fig. 5D). Consistent with this, the physical interaction between Hel2 and its ribosomal anchor RACK1/Asc1 was unaffected by the eS31 mutations in a split‐TRP1 assay (Fig. 6), a method we previously developed for this purpose [43].

**Fig. 6:**
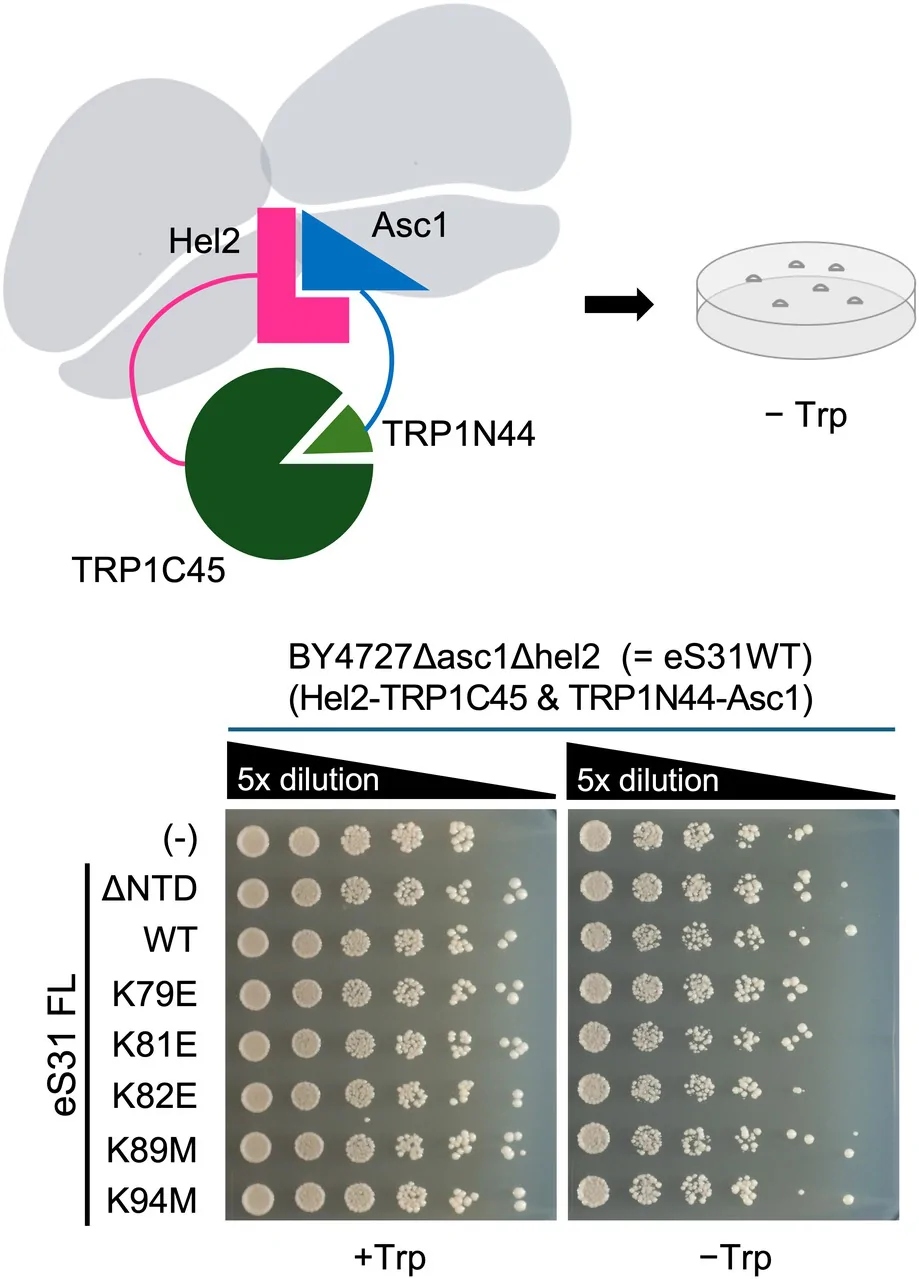
eS31 NTD mutations do not affect the Hel2–Asc1 interaction. (Top) Schematic of the split‐TRP1 reporter system. Interaction between Hel2 and Asc1, fused to complementary fragments of the TRP1 enzyme, reconstitutes tryptophan synthesis and permits growth on medium lacking tryptophan. Schematic reproduced from [43]. (Bottom) Growth assay of strains co‐expressing the Hel2–TRP1C45 and Asc1–TRP1N44 fusions, along with plasmids expressing the indicated eS31 variants. The robust growth on –Trp medium was unaffected by any of the eS31 NTD mutants, indicating they do not disrupt the physical proximity of Hel2 and Asc1 on the ribosome. Yeast strains expressing the indicated eS31 variants were serially diluted 5‐fold and spotted onto SC plates. The first spot corresponds to the undiluted culture (adjusted to OD_600_ = 0.5). Growth assays shown are representative of at least three independent transformants, which yielded comparable results.

Collectively, these genetic tests demonstrate that the eS31 NTD mutations do not act by impairing the canonical downstream machinery of NSD or NGD. The logical conclusion is that they must function upstream of these quality‐control checkpoints. Rather than disrupting the response to a stall, the mutations appear to prevent the long‐lived stall that initiates the response. Our finding that eS31 NTD mutations act upstream of quality‐control checkpoints led to the hypothesis that they alleviate stalling by directly reducing decoding fidelity.

### The stalling‐bypass phenotype is caused by reduced decoding fidelity

To test this, we converged three independent lines of evidence. First, we examined sensitivity to paromomycin, an aminoglycoside antibiotic that binds to the ribosomal A‐site and induces misreading [50, 51, 52, 53]. Consistent with a fidelity defect, strains carrying charge‐reversing mutations within the K79–K82 hotspot, whether present singly or in combination, exhibited a distinct increase in paromomycin sensitivity, while the eS31ΔNTD mutant displayed a more pronounced effect (Fig. 7A). Second, for more direct evidence, we employed a dual‐luciferase reporter that quantifies amino acid misincorporation [54, 55]. As hypothesized, the K81E and K82E mutants exhibited elevated misincorporation rates, providing direct evidence that their stalling bypass correlates with decreased translational accuracy (Fig. 7B). As a positive control for this assay, we confirmed that treatment with paromomycin also significantly increased the misincorporation rate in wild‐type BY4727 cells (Fig. 7B, right panel). These fidelity defects mirrored their paromomycin sensitivity and CGA‐bypass phenotypes (Fig. 4A). Finally, we investigated the genetic interaction with the diphthamide modification of eEF2, a crucial modification for maintaining the reading frame. We reasoned that if the mutants' primary effect is on decoding, their phenotype should be sensitive to perturbations in translocation. Indeed, in a *dph2Δ* background, which lacks diphthamide and has a high rate of −1 frameshifting [56, 57], the growth phenotypes of the eS31 NTD point mutants were completely suppressed (Fig. 7D). This powerful genetic suppression supports a model where the catastrophic frameshifting from the loss of diphthamide is a dominant defect that masks the subtler fidelity‐tuning contribution of the eS31 NTD.

**Fig. 7:**
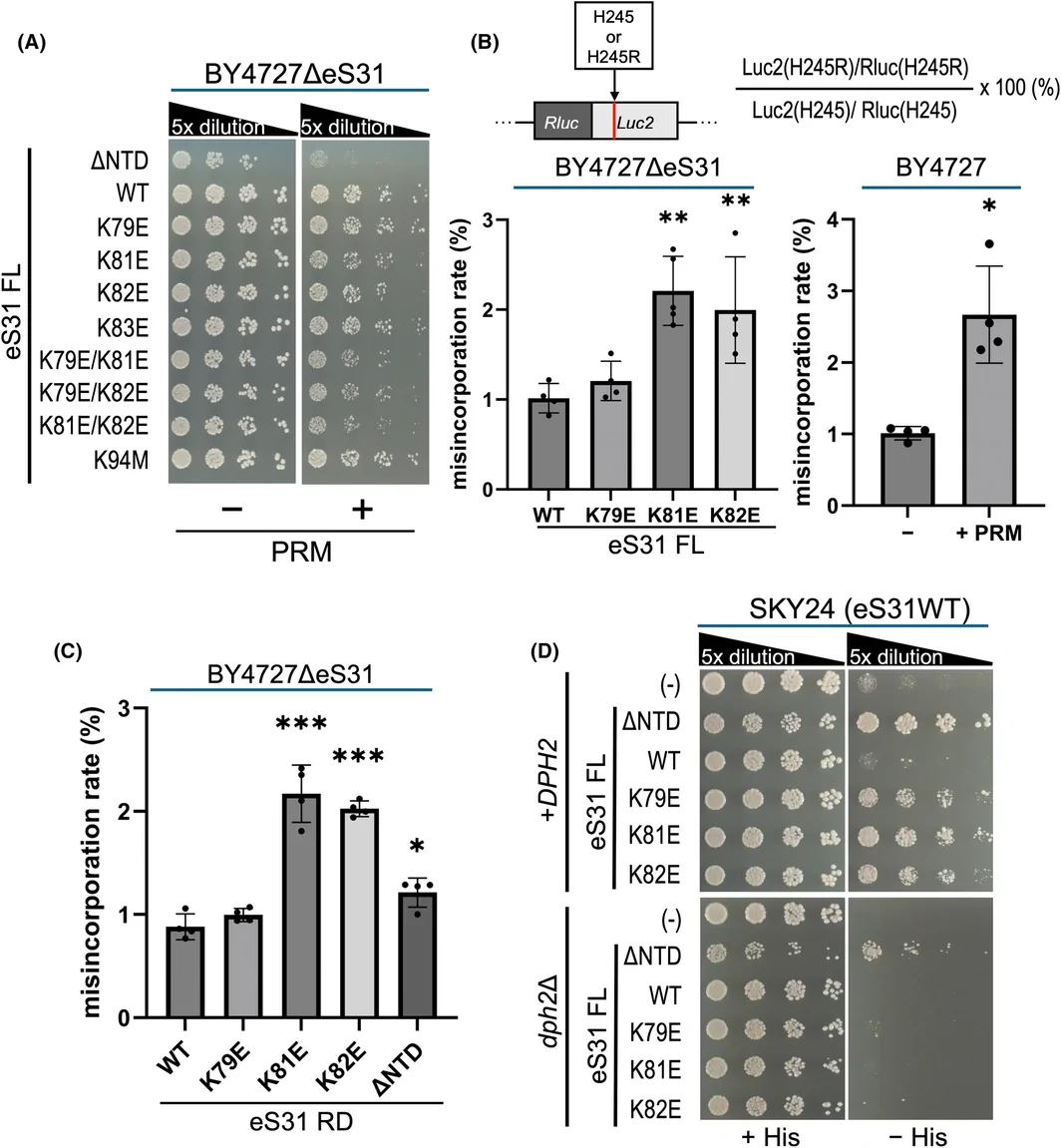
Key lysines in the eS31 NTD contribute to decoding fidelity. (A) eS31 NTD mutations confer hypersensitivity to the misreading‐inducing antibiotic paromomycin. *UBI3* knockout strains expressing the indicated eS31 variants were spotted on medium with or without paromomycin (2 mg·mL^−1^). Paromomycin (PRM). (B) eS31 NTD mutations directly increase amino acid misincorporation. (Top) Schematic of the dual‐luciferase reporter that quantifies misreading of a near‐cognate codon. (Bottom) Misincorporation rates for the indicated eS31 variants in BY4727ΔeS31 strain (left graph) and for wild‐type BY4727 cells treated with (+) or without (−) paromomycin (right graph). The paromomycin treatment serves as a positive control, confirming the assay's ability to detect misreading. Data are shown as mean ± SD. Each dot represents an independent biological replicate (*n* = 4–5 per condition, as indicated). Statistical significance was assessed by one‐way ANOVA with Dunnett's post‐test compared to WT (*P < 0.05, **P < 0.01) (left) and with paired two‐tailed *t*‐test (right). (C) Misincorporation reporter assay for eS31 RD variants, including ΔNTD. Misincorporation rates were measured in *UBI3* knockout strains expressing ribosome‐domain (RD) only versions of the indicated eS31 variants. The results closely parallel those obtained with the full‐length eS31 constructs (B), confirming that the observed fidelity defects are an intrinsic property of the eS31 NTD and not an artifact of the fused ubiquitin moiety. Data are shown as mean ± SD. Each dot represents an independent biological replicate (*n* = 4). Statistical significance was assessed by one‐way ANOVA with Dunnett's post‐test compared to WT (*P < 0.05, ***P < 0.001). (D) The eS31 mutant phenotype requires an eEF2 diphthamide modification. Growth assay of a *dph2Δ* CGA_12_–*HIS3* reporter strain, which lacks the eEF2 diphthamide modification. The growth phenotype of the point mutants is suppressed in the *dph2Δ* background but is restored upon reintroduction of a wild‐type *DPH2* plasmid (*DPH* ^+^). Yeast strains expressing the indicated eS31 variants were serially diluted 5‐fold and spotted onto SC plates. The first spot corresponds to the 10^−1^ dilution of the normalized culture (OD_600_ = 0.1) in (A) and (D). Growth assays shown in (A) and (D) are representative of at least three independent transformants with comparable results.

Taken together, these data converge on a single conclusion: the K79–K82 cluster of the eS31 NTD is a key determinant of decoding fidelity. By shaping the A‐site's electrostatic environment, these basic residues enforce stringent codon–anticodon recognition. Reversing their charge compromises this stringency, leading to the misreading that allows ribosomes to bypass the CGA stall in our reporter system.

### Complete NTD deletion reveals a distinct mechanism involving impaired termination fidelity

While both point mutations and the complete deletion (eS31ΔNTD) alleviate CGA‐induced stalling, compelling evidence indicated they operate through fundamentally distinct mechanisms. The eS31ΔNTD mutant presented a paradox: it was hypersensitive to paromomycin yet showed no increase in amino acid misincorporation (Fig. 7A,C). Furthermore, in stark contrast to the point mutants, the stalling‐bypass phenotype of eS31ΔNTD was completely unaffected by the loss of the eEF2 diphthamide modification (*dph2Δ* strain) (Fig. 7D). These results strongly suggested that the eS31ΔNTD phenotype is not driven by a simple defect in sense codon decoding.

This striking divergence prompted us to measure translation termination efficiency using a dual‐luciferase reporter for premature UGA stop codon readthrough (Fig. 8A). The result was compelling: eS31ΔNTD induced a pronounced, approximately fourfold increase in stop codon readthrough, a severe termination fidelity defect not observed with any of the point mutants (Fig. 8B). This finding provides a direct mechanistic explanation for the unique eS31ΔNTD phenotype and establishes a clear double dissociation between the two functional modes of the NTD. Specifically, while the K79–K82 point mutations increase misincorporation (elongation) without affecting readthrough (termination), the ΔNTD deletion impairs termination fidelity without increasing misincorporation rates. This distinguishes the effect of the full deletion as a global structural perturbation that specifically affects termination, in contrast to the residue‐specific modulation of decoding stringency.

**Fig. 8:**
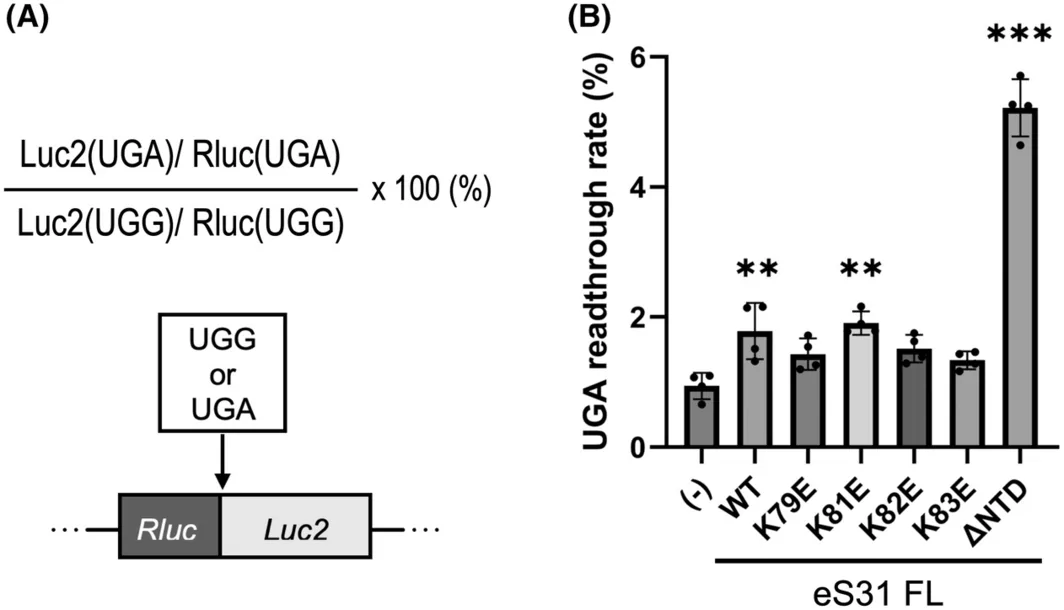
Complete deletion of the eS31 NTD causes a severe termination fidelity defect. (A) Schematic of the dual‐luciferase reporter used to measure readthrough of a premature UGA stop codon. Readthrough efficiency is calculated as the normalized Luc2/Rluc ratio from the UGA reporter relative to a UGG (sense codon) control, indicated as the formula above. (B) eS31ΔNTD, but not the NTD point mutants, significantly increase stop codon readthrough. Assays were performed in the *UBI3* ^+^ background. Data are shown as mean ± SD. Each dot represents an independent biological replicate (*n* = 4). Statistical significance was assessed by one‐way ANOVA with Dunnett's post‐test compared to empty control (**P < 0.01, ***P < 0.001).

It is worth noting that the regulatory impact of ribosomal mutations extends across all stages of the translation cycle. For instance, specific mutations in 18S and 25S rRNA have been demonstrated to increase the stringency of start codon selection and suppress translation initiation from non‐AUG codons [58, 59]. Taken together, our findings regarding the role of the eS31 NTD in elongation and termination fidelity complement these observations, suggesting that different components of the ribosome—both protein and RNA—collectively ensure the overall accuracy of protein synthesis.

### The eS31ΔNTD phenotype paradoxically requires wild‐type eS31 and implicates ribosome heterogeneity

Our analysis of the eS31ΔNTD mutant revealed a striking paradox that challenged the principles of a dominant‐negative phenotype. Unlike the NTD point mutants, which conferred a stalling‐bypass phenotype both in the presence and absence of chromosomal *UBI3*, the eS31ΔNTD phenotype was robust only when co‐expressed with wild‐type eS31. When eS31ΔNTD was the sole variant in a *ubi3Δ* strain, the effect was almost completely abolished (Figs 3B, 9A). This suggested the phenotype is not an intrinsic property of eS31ΔNTD‐containing ribosomes.

**Fig. 9:**
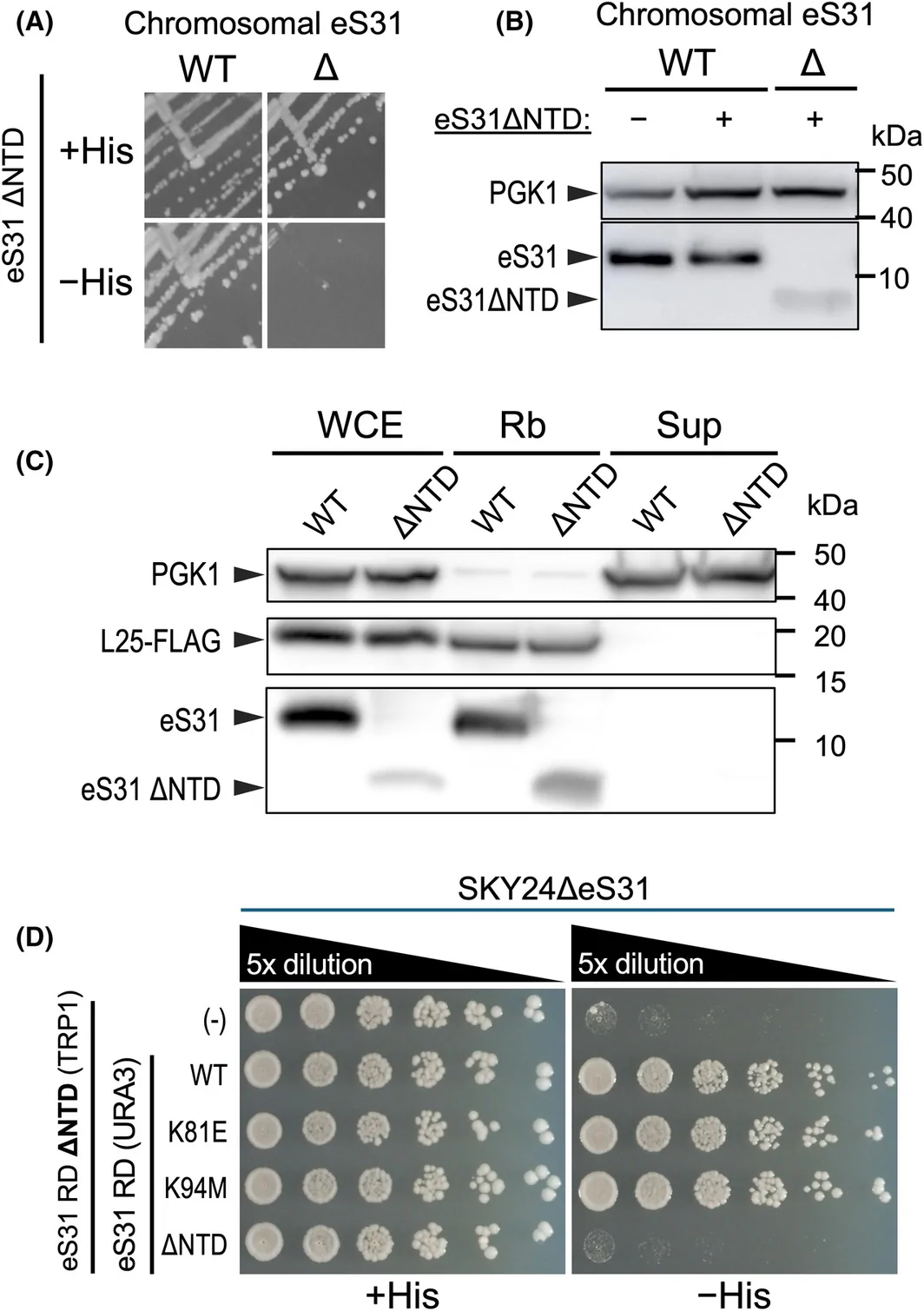
The dominant‐negative effect of eS31ΔNTD is lost when it is the sole source of eS31. (A) Growth assay of the CGA_12_–*HIS3* reporter strain expressing eS31ΔNTD in a background with the endogenous *UBI3* gene present (WT: *UBI3* ^+^) or deleted (Δ: *ubi3Δ*). Growth on –His medium is observed only in the *UBI3* ^+^ background. Images represent cropped sections from standard 12 cm agar plates. Full uncropped plate images are available in Fig. S1. The left panels (WT) and the rightmost panels in Fig. 1D display slightly different cropped regions from the same original plate image. This reuse is intentional to facilitate direct visual comparison. (B) eS31ΔNTD incorporation is dependent on the genetic background. Western blot analysis of eS31 levels in the strains from (A). (C) eS31ΔNTD is more efficiently incorporated into ribosomes in the *ubi3Δ* background. Western blot analysis of whole‐cell extracts (WCE), ribosome pellets (Rb), and post‐centrifugation supernatants (Sup) from *ubi3Δ* strains expressing either wild‐type eS31 or eS31ΔNTD as the sole source. PGK1 served as a cytoplasmic control, and L25‐FLAG as a ribosomal marker. (D) Co‐expression of wild‐type eS31 rescues the eS31ΔNTD phenotype in the *ubi3Δ* background. A *ubi3Δ* strain expressing eS31ΔNTD from Plasmid 1 (*TRP1*) was co‐transformed with Plasmid 2 (*URA3*) carrying the indicated eS31 variants. Growth on –His medium was restored only when additional eS31 (WT or point mutant) was co‐expressed. Yeast strains expressing the indicated eS31 variants were serially diluted 5‐fold and spotted onto SC plates. The first spot corresponds to the undiluted culture (adjusted to OD_600_ = 0.5). Growth assays shown are representative of at least three independent transformants tested in parallel, all of which exhibited reproducible phenotypes. Western blot results are representative of at least three independent biological experiments performed using independent transformants.

This paradox was not due to trivial effects on protein expression or incorporation. In the presence of wild‐type eS31, the truncated protein was incorporated at very low levels, yet this was sufficient for a strong phenotype (Fig. 9B). Conversely, in the *ubi3Δ* background where the phenotype was lost, eS31ΔNTD was robustly incorporated into ribosomes (Fig. 9C). To test this “trans‐acting” disturbance model directly, we performed a plasmid add‐back experiment. In the *ubi3Δ* background, where eS31ΔNTD alone confers no phenotype, co‐expression of wild‐type eS31 from a second plasmid fully restored the strong stalling‐bypass effect (Fig. 9D).

These findings reveal a profound mechanism. The eS31ΔNTD does not simply function on the ribosome it occupies; its presence, even at low levels of incorporation, appears to cause a global, trans‐acting disruption that alters the function of the major wild‐type ribosome population. This provides a compelling *in vivo* model for the emerging paradigm of ribosome heterogeneity, suggesting that compositionally distinct ribosome pools can dynamically regulate cellular translation.

### A short C‐terminal segment of the NTD is critical to prevent the ΔNTD phenotype

To map the structural requirements for the paradoxical ΔNTD phenotype, we constructed a series of stepwise N‐terminal truncations (Fig. 10A). When tested in the dominant‐negative assay, the resulting stalling‐bypass phenotype was not graded with the extent of the deletion. Instead, the effect appeared abruptly and only upon complete removal of the NTD (Δ77–99); as strikingly, none of the partial truncations were active (Fig. 10B).

**Fig. 10:**
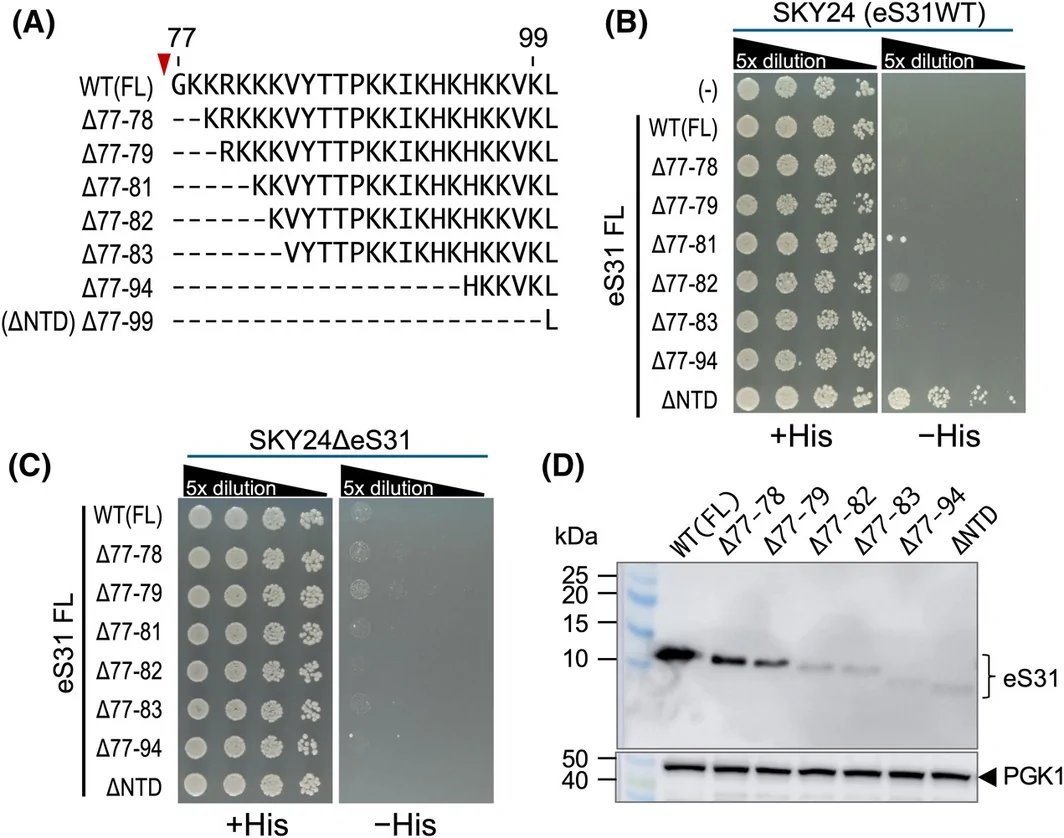
A short N‐terminal segment of the NTD acts as a molecular switch for the ΔNTD phenotype. (A) Schematic of the stepwise N‐terminal truncations. A series of deletion mutants were constructed, starting from residue G77. The deleted residues are indicated by dashes. Cleavage site of ubiquitin moiety is shown by a red arrow. (B) Growth assay of the eS31 N‐terminal truncation series in the CGA_12_–*HIS3* reporter strain (SKY24, UBI3^+^ background). While all variants grew on the control plate containing histidine (+His), growth on the selective plate lacking histidine (–His) was observed only with the complete deletion of the NTD (Δ77–99), indicating that the stalling‐bypass phenotype is only triggered upon complete removal of the NTD. (C) Growth assay of the eS31 N‐terminal truncation series in the CGA_12_–*HIS3* reporter strain (SKY24ΔeS31, *ubi3Δ* background). While all variants grew on the control plate containing histidine (+His), none of the deletion mutants (including Δ77–99; ΔNTD) showed growth on the selective plate lacking histidine (–His). Growth test demonstrates that all constructs can function as the sole source of eS31, indicating that the stalling‐bypass phenotype of ΔNTD is only triggered in the presence of wild‐type eS31 on the genome. The first spot corresponds to the 10^−1^ dilution of the normalized culture (OD_600_ = 0.1) in (B) and (C). (D) Western blot analysis of strains from (C) confirms the expression of progressively shorter eS31 proteins. PGK1 served as a loading control. Growth assay images and western blot results are representative of at least three independent biological experiments performed using independent transformants.

This abrupt appearance of the phenotype was not due to a lack of expression or viability, as all truncation mutants—including the full ΔNTD—supported cell viability as the sole source of eS31 and produced stable proteins of the expected sizes (Fig. 10C,D). This analysis reveals an abrupt phenotypic transition between the inactive Δ77‐94 deletion mutant and the fully active Δ77‐99 deletion mutant, pinpointing the five C‐terminal‐most amino acids (HKKVK) as the functional boundary. The all‐or‐nothing nature of this transition strongly suggests that this HKKVK segment acts as a structural switch or anchor, rather than a graded regulatory motif. Its removal likely compromises the structural integrity of the entire domain, triggering the global translational disruption and termination defects characteristic of the NTD phenotype.

## Discussion

### Dissecting the eS31 NTD: Two distinct functional modes

Our high‐resolution genetic screen, bypassing the pleiotropic artifacts of domain deletion, dissected the NTD's role in translational control into two functionally and mechanistically distinct modes. The first is a residue‐specific, electrostatic mechanism for fine‐tuning decoding fidelity, as revealed by our analysis of point mutants (Fig. 7). The second is a broader structural role for the entire domain in maintaining the integrity of the decoding center, which is critical for high‐fidelity termination, a function uncovered through the paradoxical behavior of the complete deletion mutant (eS31ΔNTD) (Figs 8, 9, 10). Collectively, our findings not only clarify the function of eS31 but also provide a compelling model for how ribosome heterogeneity can impact global translation dynamics.

### An electrostatic hotspot in the eS31 NTD fine‐tunes translational accuracy

Our work identifies a functional hotspot within the eS31 NTD—a cluster of basic residues (K79–K82)—that modulates A‐site decoding. This positively charged hotspot shapes the A‐site's local electrostatic environment, enforcing a stringent checkpoint for codon–anticodon pairing. Consequently, neutralizing or reversing this charge lowers the fidelity threshold, providing a clear mechanistic basis for bypassing the CGA stall before RQC is triggered. The hotspot thus functions not as part of the rescue machinery, but as a fidelity modulator that determines whether a pause escalates into a stall requiring rescue. The nature of the CGA stall, which arises from a direct structural impediment hindering any tRNA entry, suggests a nuanced bypass mechanism [35].

The compromised stringency in our mutants may therefore act in several non‐mutually exclusive ways: by lowering the acceptance barrier for near‐cognate tRNA, by electrostatically accommodating the hindered cognate tRNA, or by tolerating an imperfect codon‐anticodon interaction within the distorted A‐site. In any scenario, the fundamental effect is a lowered activation energy barrier for tRNA accommodation at this problematic codon.

### A structural basis for the dual roles of the eS31 NTD in translational fidelity

Our findings provide compelling functional evidence for structural predictions from recent cryo‐EM studies, establishing the eS31 NTD as a versatile interaction hub that enforces translational accuracy at two distinct stages: termination and elongation. As a flexible extension projecting from the 40S beak domain with its charged tip dipping into the mRNA path, the NTD is perfectly positioned to modulate a wide range of factors that occupy the ribosomal A‐site.

The severe termination defect of the ΔNTD mutant (Fig. 8B) is likely explained by the NTD's critical interaction with the termination factor eRF1 [60]. Intriguingly, our charge‐reversing point mutations had only a modest effect on termination (Fig. 8B), suggesting that the interaction is not mediated by a simple electrostatic attraction. Instead, high‐fidelity termination likely relies on the structural integrity of the entire NTD acting as a physical scaffold for the proper positioning of eRF1. This model is strongly supported by our previous genetic screen, which isolated mutations in eRF1 that suppressed termination defects [61]. Remarkably, these mutations map to two distinct regions of eRF1—the N‐terminal lobe (E100K) and the mini‐domain (Q359R, E360G, E366G)—both of which are positioned in close proximity to the eS31 NTD in structural models, with the first two mini‐domain mutations being particularly adjacent to the upstream basic residue cluster of the NTD (Fig. 11). This leads to a compelling new model where eRF1 utilizes a “multiple‐point grip” to clamp the NTD, rigidly anchoring it in the correct conformation for high‐fidelity stop codon recognition. The complete loss of the NTD would abolish these critical contact points, providing a direct structural explanation for the severe termination defect we observed. This resolves a key ambiguity from previous studies: the fidelity defects of eS31ΔNTD are not simply due to the loss of a specific regulatory surface but reflect a profound structural destabilization of the decoding center that is critical for accurate termination.

**Fig. 11:**
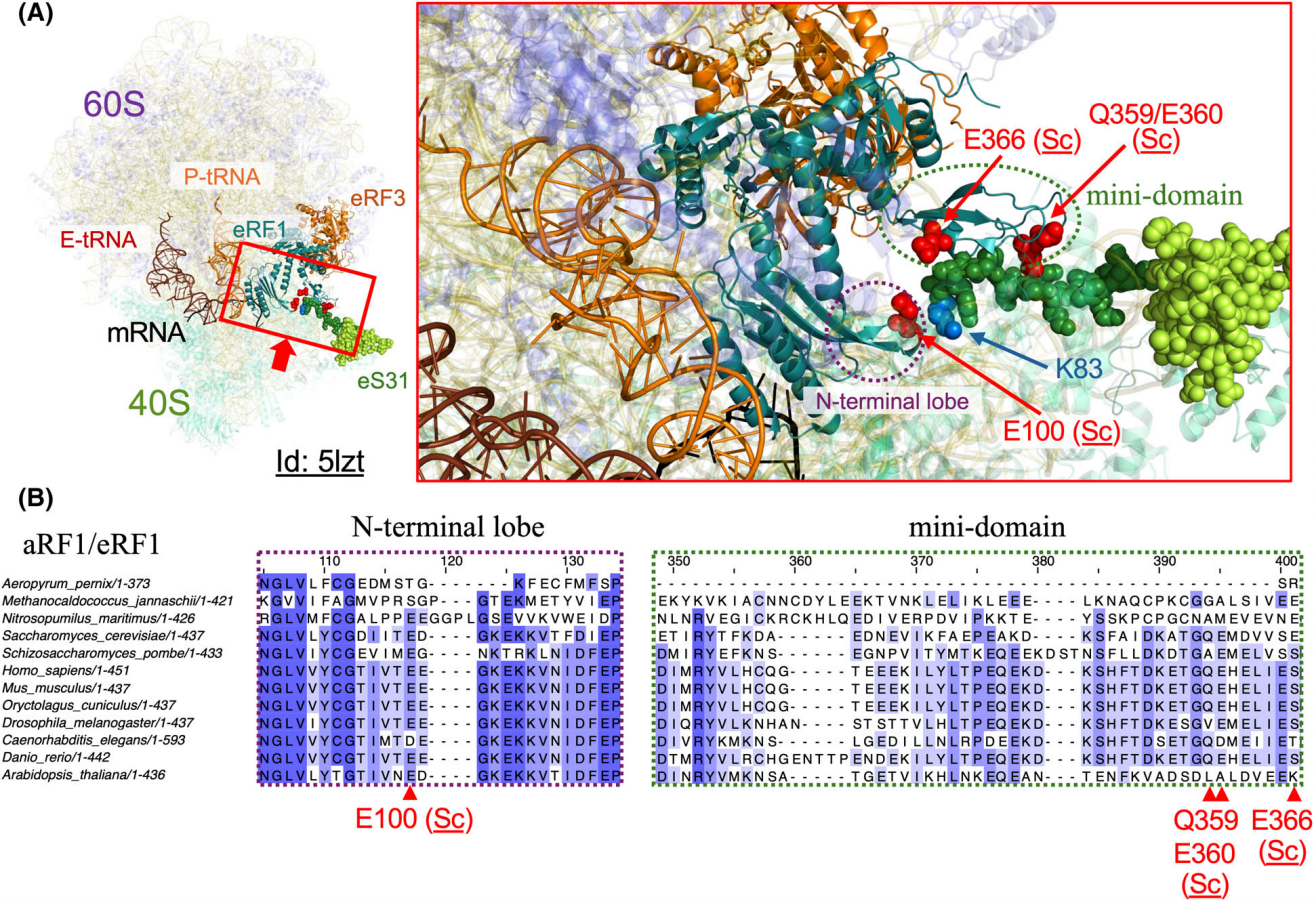
Structural model of the eRF1–eS31 NTD interaction. (A) A structural model of the mammalian 80S ribosome in a termination‐competent state (based on pdb id: 5lzt) highlights the interaction between eRF1 and the eS31 NTD. The overall view (left) shows eRF1 (domains colored) and eS31 (green) at the decoding center. The magnified view (right) illustrates the proposed “three‐point grip” interaction [24]. Residue E100 in the N‐terminal lobe of eRF1 is in close proximity to the eS31 NTD hotspot (K79–K82), while residues Q359, E360, and E366 in the mini‐domain are positioned to interact with another surface of the NTD. All the eRF1 residues are in *S. cerevisiae* numbering. (B) Sequence alignment of eRF1 orthologs focusing on the N‐terminal lobe and mini‐domain. The conserved residues E100, Q359, E360, and E366 (*S. cerevisiae* numbering) are highlighted. Species are ordered from Archaea (*Aeropyrum pernix* [WP_131159543], *Methanocaldococcus jannaschii* [WP_244409515], *Nitrosopumilus maritimus SCM1* [ABX12778]) to Eukarya (*Saccharomyces cerevisiae* [NP_009701], *Schizosaccharomyces pombe* [CAB75769], *Homo sapiens* [P62495], *Mus musculus* [NP_659115.3], *Oryctolagus cuniculus* [NP_001076236], *Drosophila melanogaster* [NP_001262104], *Caenorhabditis elegans* [NP_001343671], *Danio rerio* [NP_958868], *Arabidopsis thaliana* [NP_001032029]).

A similar structural interplay governs the NTD's role in elongation fidelity. Our analysis provides a mechanistic basis for the potent genetic interaction observed between the hotspot mutants and the loss of eEF2's diphthamide modification (*dph2Δ* background, Fig. 7D). In a structure capturing eEF2 during translocation, the NTD hotspot (K79**–**K82) is positioned directly behind the crucial diphthamide loop of eEF2 (Fig. 12) [23]. The charge reversal in our mutants would disrupt this interaction, likely destabilizing eEF2 and thus explaining the observed misreading and potent genetic suppression by *dph2Δ*.

**Fig. 12:**
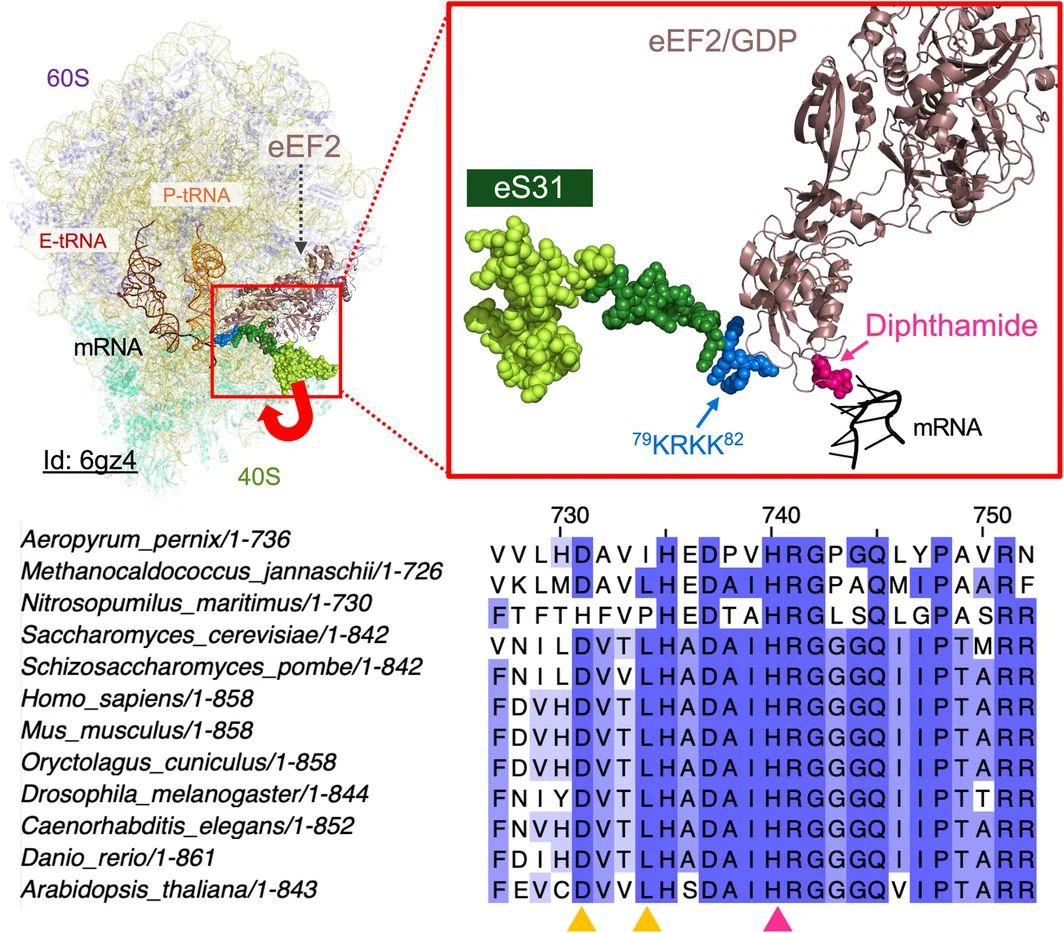
Structural model of the eEF2–eS31 NTD interaction. (upper) A structural model of the mammalian 80S ribosome with eEF2 captured in a translocation‐intermediate‐POST‐2 state (based on pdb id: 6gz4) [23]. The overall view (left) and a magnified view of the A‐site (right) are shown. The diphthamide‐modified histidine on eEF2 that contacts the mRNA is colored pink. The eS31 NTD functional hotspot (residues K79–K82) is colored blue, showing its close proximity to the diphthamide loop of eEF2 from just behind. (lower) Sequence alignment of eEF2 orthologs using MEGA and Jalview software [62]. Residues on eEF2 predicted to be within 4 Å of the eS31 hotspot are indicated by yellow arrows. The conserved histidine residue that undergoes diphthamide modification is indicated by a pink arrow. The species included in the alignment are the same as in Fig. 11. (*Aeropyrum pernix* [Q9YC19], *Methanocaldococcus jannaschii* [WP_010870561], *Nitrosopumilus maritimus* [WP_012215131], *Saccharomyces cerevisiae* [P32324], *Schizosaccharomyces pombe* [CAB58373], *Homo sapiens* [NP_001952], *Mus musculus* [NP_031933], *Oryctolagus cuniculus* [XP_051684885], *Drosophila melanogaster* [NP_724358], *Caenorhabditis elegans* [NP_001369996], *Danio rerio* [XP_697966], *Arabidopsis thaliana* [KAL9860265]).

A major challenge in visualizing these potential interactions is the highly flexible nature of the NTD, which has precluded the capture of its full‐length structure in most cryo‐EM reconstructions. Nevertheless, a superposition of the available eS31 structures from different translational states reveals a key insight: while the ribosome undergoes global conformational changes, a large portion of the eS31 remains remarkably stable, with significant flexibility confined to the distal tip of NTD where the functional hotspot we identified resides (Fig. 13A). This underlying stability provides a framework for predictive modeling. By superimposing the longest resolved NTD structure (pdb id: 5lyb) onto various models of the ribosome complexed with A‐site factors, we can predict specific points of contact (Fig. 13B). Thus, structural modeling predicts a direct electrostatic interaction (within 4 Å) between the positively charged hotspot and the phosphate backbone of the A‐site tRNA anticodon loop (Fig. 13C). This interaction could stabilize the codon‐anticodon helix to enforce stringent decoding, a process known to be sensitive to anticodon loop modifications [63].

**Fig. 13:**
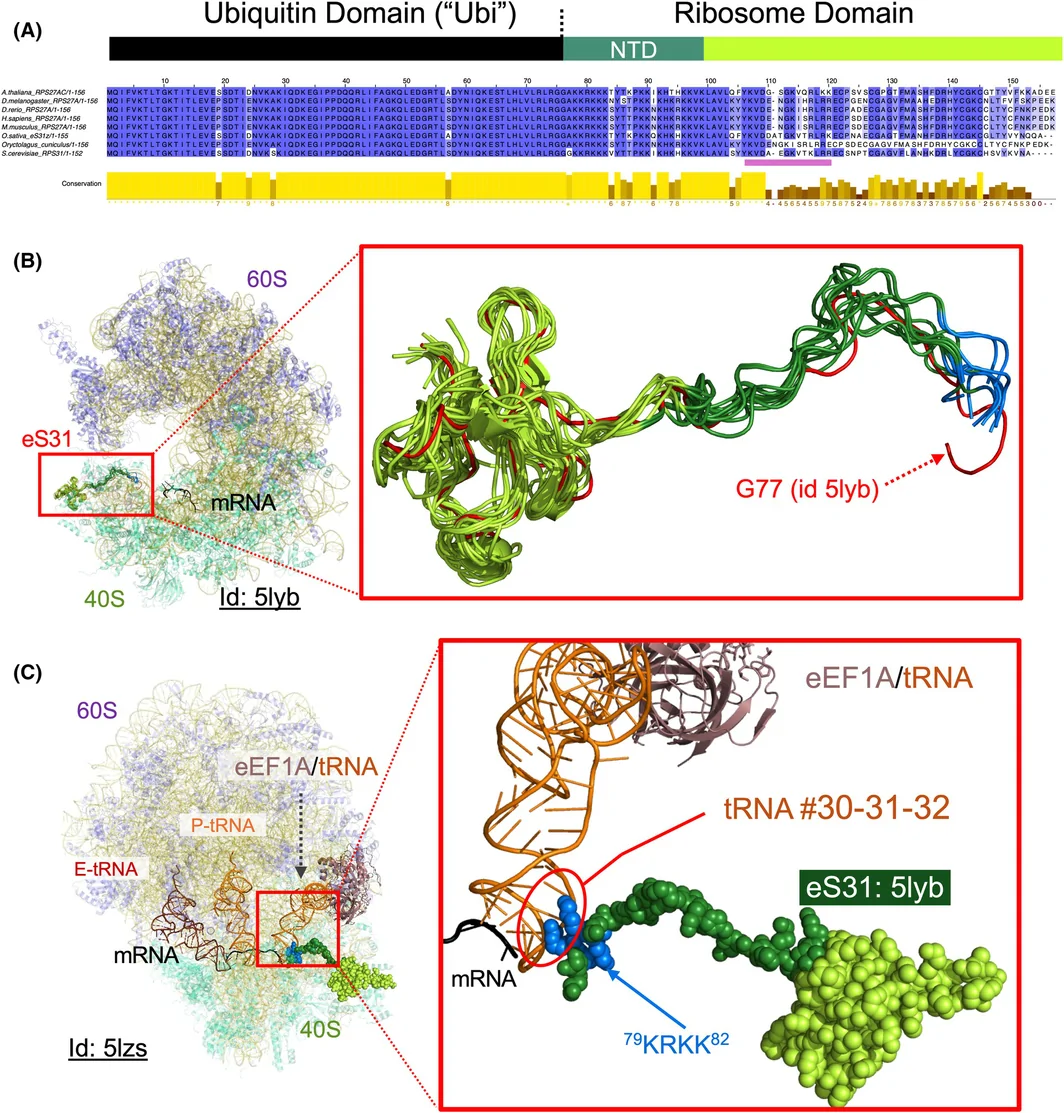
Structural flexibility and superposition of the eS31 NTD. (A) Sequence alignment of eS31 orthologs. A schematic representation of the eS31 protein domains is shown above the alignment. eS31 protein sequences from the indicated species (*A. Thaliana* [NP_173755]), *D. melanogaster* [AAF52941], *D. rerio* [NP_956796], *H. sapiens* [NP_002945], *M. musculus* [P62983], *O. sativa* [NP_001395951], *Oryctolagus cuniculus* [XP_069925205], and *S. cerevisiae* [NP_013268]) were aligned using MEGA and Jalview software [62]. The degree of amino acid conservation across the orthologs is indicated in the “Conservation” track below the alignment. The peptide sequence used to raise the custom anti‐eS31 antibody used in this study is indicated by the pink bar. (B) Superimposition of eS31 structures from various ribosomal states, shown in cartoon representation. All structures were aligned based on the globular domain of eS31 and are displayed on the ribosome from the 5lyb structure [64]. This comparison reveals that the overall structure of eS31 is largely conserved, irrespective of the ribosome's translational state, with the notable exception of the tip of its N‐terminal domain (NTD). The eS31 from the 5lyb structure, which possesses the most extended NTD in a ribosome with a vacant A‐site, is highlighted in red. In the 5lyb structure, the terminal residue, G77, is resolved. PDB IDs used for the analysis: *Yeast*: 3j77 (post‐translocation‐like state), 3j78 (translocating state) [65], 5lyb (Pre‐Peptidyltransfer state) [64], 5m1j (DOM34 bound state) [26], 6t4q (CGA‐CCG stall ribosome), 6t7t (poly(A) stall ribosome) [35], 8z70 (mRNA decoding, State S1d), 8z71 (mRNA decoding, State S1a), 8xu8 (peptidyl transfer, State S2c, with eEF2), 8yld (tRNA translocation, State S4a, with eEF2), 8ylr (tRNA translocation, State S6, with eEF2) [66]. *Rabbit*: 5lzs (tRNA Codon recognition with eEF1A), 5lzt (eRF1 codon recognition), 5lzu (eRF1 accommodating state), 5lzv (eRF1 recycling state) [24]. (C) Predicted interaction between the eS31 NTD and the A‐site tRNA during decoding. The full‐length eS31 NTD structure (from pdb: 5lyb) [64] was superimposed onto a ribosome structure capturing the decoding state with eEF1A and a cognate tRNA in the A‐site (pdb: 5lzs). The overall view (left) and a magnified view of the decoding center (right) are shown. The model predicts that the eS31 hotspot (K79–K82, blue) is positioned within 4 Å of the phosphate backbone of the A‐site tRNA (near residues 30–32), suggesting a direct electrostatic interaction.

Collectively, these insights portray the eS31 NTD as a sophisticated interaction hub, using its flexible, charged tip to engage with diverse A‐site ligands—eRF1, eEF2, and tRNA—and enforce translational accuracy at multiple critical stages of protein synthesis. These observations emphasize a clear functional distinction between the residue‐specific role of the K79–K82 cluster and the broader structural contribution of the complete NTD. Although these residues are situated near the NLS, the robust growth and wild‐type‐like incorporation levels of the hotspot mutants (Figs 2 and 3) indicate that nuclear import and ribosome biogenesis remain functionally intact. Rather than causing the general translational impairment typical of assembly failures, these mutations specifically modulate decoding stringency at the elongation stage. This reinforces a double dissociation model where the K79–K82 cluster primarily governs elongation fidelity, whereas the overall integrity of the NTD is essential for accurate termination.

### The ΔNTD paradox: A model for trans‐acting disruption and ribosome heterogeneity

One of the most profound findings of this study is the paradoxical genetic behavior of the eS31ΔNTD mutant. Its stalling‐bypass phenotype is not a cell‐autonomous property of the mutant ribosome itself; rather, it manifests only in a heterogeneous population where wild‐type eS31 is also present (Fig. 9A,C). This leads to a startling conclusion: a minor sub‐population of structurally compromised ribosomes, incorporated at very low levels (Fig. 9B), is sufficient to cause a global disruption that alters the function of the wider, wild‐type ribosome population.

This “trans‐acting” disruption provides a compelling *in vivo* model for the emerging paradigm of ribosome heterogeneity [67], but suggests a mechanism distinct from a prevailing model, such as “specialized ribosomes” promoting preferential translation of specific subsets of mRNAs. In contrast, our findings suggest a “trans‐acting disruption” model. Here, the eS31ΔNTD phenotype arises not from the selective translation of specific mRNAs by specialized ribosomes, but from a global perturbation of the entire cellular ribosome pool. How this disruption is propagated—whether through competition for limiting translation factors, titration of quality‐control components, or other unforeseen mechanisms—remains an exciting avenue for future investigation.

This model may also provide insight into the pathology of human ribosomopathies, such as Diamond‐Blackfan Anemia, where haploinsufficiency of a single ribosomal protein gene leads to tissue‐specific defects despite the presence of a wild‐type allele [68]. The disease phenotype may not arise solely from the reduced number of functional ribosomes, but from a dominant‐negative or trans‐acting disruption of proteostasis caused by the incorporation of faulty components, for which our eS31ΔNTD system provides a powerful and tractable yeast model. Ultimately, these findings position the eS31ΔNTD mutant not just as a tool for studying eS31, but as a unique platform for exploring how compositional diversity within the ribosome pool can dynamically regulate cellular proteostasis.

In conclusion, our work establishes the eS31ΔNTD as a sophisticated, bifunctional regulatory element, rather than a structurally uncharacterized extension. Our findings reveal a new layer of translational control specific to eukaryotes, where a single protein employs distinct mechanisms—local electrostatics to maintain elongation fidelity and global structural integrity to ensure termination accuracy—drawing a compelling parallel to the classical paradigms of translation fidelity established in bacteria [69, 70, 71]. This evolutionary innovation underscores the complexity of the eukaryotic ribosomal beak, which, unlike its simpler bacterial counterpart, has incorporated additional protein elements like eS31 to create a more versatile regulatory hub [60, 72]. This work not only provides a definitive, high‐resolution view of eS31 function but also opens up new avenues for understanding the profound impact of ribosome diversity on gene expression and cellular health.

## Materials and methods

### Yeast strains and medium

*Saccharomyces cerevisiae* strains used in this study are listed in Table S1 [27, 41, 43, 48, 73]. Reporter strains carrying integrated CGA_12_–*HIS3* or *Rluc–luc2* constructs were generated by homologous recombination at the *HO* locus. Yeast cells were cultured in YPD or synthetic complete (SC) medium supplemented with the appropriate dropout mix (ForMedium, Hunstanton, UK), with 2% agar added for plates. For plasmid maintenance, cells were grown on SC plates lacking the relevant auxotrophic marker(s). Although eS31 (encoded by *UBI3*) is sometimes described as non‐essential in certain yeast genetic backgrounds, it is categorized as essential for viability in the S288C‐derived background [74]. We confirmed that *ubi3Δ* is strictly inviable in our BY4727 strain (S288C derivative) through plasmid shuffling assays (Fig. S2). To generate *UBI3* knockout (ΔeS31) strains, yeast cells were first transformed with a p416GPD‐based plasmid expressing wild‐type *UBI3* (p416GPD‐eS31). The endogenous *UBI3* locus was then replaced by the *K. lactis* LEU2 marker from pUG73 [75]. Replacement of the p416GPD‐eS31 with p414GPD‐based plasmids expressing eS31 variants was achieved by plasmid shuffling on SC plates containing 5‐fluoroorotic acid (5‐FOA, 0.6 g·L^−1^) as previously described [76]. Experiments were conducted in either *UBI3* knockout or dominant‐negative backgrounds, depending on viability and feasibility; both yielded consistent trends, ensuring comparable interpretation.

For growth assay, yeast strains were spotted or streaked on plates and incubated at 30 °C for 3–5 days. Images shown are representative of at least three independent transformants. For results of streaking assays, panels shown in the figure were cropped from the same plate for clarity. Original, uncropped images are presented in Fig. S1.

### Growth and spotting assays

For spotting assays, yeast cells were grown overnight in the appropriate selective liquid medium at 30 °C, diluted into fresh medium, and cultured to log phase. Cultures were then washed with sterile water, adjusted to an equal OD_600_, and serially diluted 5‐fold. Aliquots of each dilution were spotted onto the indicated plates. The starting concentration for each assay (e.g., undiluted culture or a specific dilution thereof) is specified in the relevant figure legend. Plates were incubated at 30 °C for 3–5 days before being photographed.

### Plasmid construction

Throughout this study, the designation ‘WT’ (wild‐type) refers to a construct with an otherwise unaltered amino acid sequence. For deletion constructs such as ΔNTD, ‘WT’ serves as the baseline reference without any additional point mutations. Plasmids used in this study are listed in Table S2 [43, 76]. DNA fragments encoding relative protein factors were amplified by PCR from genomic DNA of the BY4727 strain using primers complementary to sequences immediately upstream and downstream of the open reading frames. Restriction sites for EcoRI (at the 5′ end) and SalI (at the 3′ end) were incorporated into the primers. PCR products were digested and cloned into the corresponding vectors linearized with the same restriction enzymes. For the misincorporation reporter, the Rluc‐Luc2 fusion construct was amplified by PCR from plasmid pHSG399‐Rluc‐Luc2 (laboratory stock) and cloned into the p413TEF vector. The H245R reporter was generated by site‐directed mutagenesis, changing codon 245 of the Luc2 gene from CAC (His) to CGC (Arg).

### Error‐prone PCR

Random mutagenesis was performed by error‐prone PCR as previously described. Briefly, DNA fragments encoding eS31 or eS31 RD were amplified with Taq polymerase under conditions that reduce its fidelity. Primer pairs used in error‐prone PCR are GPDprom (5′‐GGAAAAAGCTGAAAAAAAAGGTTG‐3′) and CYCterm (5′‐GGGACCTAGACTTCAGGTTGTC‐3′) to amplify DNA fragments encoding eS31 or eS31 RD. The p414GPD plasmid backbone was linearized with EcoRI and SalI. Amplified DNA fragments and the linearized plasmid were mixed and co‐transformed into the CGA_12_–*HIS3* reporter strain. Through homologous recombination, regenerated plasmid vectors carrying dominant‐negative mutants of eS31 or eS31 RD were obtained. Transformants were selected on SC‐Trp‐His plates and incubated at 30 °C for up to 4 days. To validate the phenotypes, candidate plasmids were recovered from yeast, amplified in *E. coli*, and reintroduced into a fresh reporter strain to confirm their growth properties. Only plasmids that reproducibly conferred growth were subjected to sequencing. For clones carrying multiple substitutions, the causative mutation was identified by individually testing single point mutations generated by site‐directed mutagenesis.

### Western blot analysis and antibodies

For analysis of ribosome assembly of eS31 variants, yeast strain Y218ΔeS31 (Table S1) was transformed with p414GPD‐eS31 variants to replace p416GPD‐eS31 using plasmid shuffling. Transformants were grown in selective medium to log phase, and cells from 10 mL cultures were harvested by centrifugation. Cell pellets were resuspended in lysis buffer [20 mm HEPES‐KOH (pH 7.5), 100 mm KCl, 5 mm MgCl_2_, 1 mm DTT, and protease inhibitor cocktail (Nacalai Tesque)] and disrupted with glass beads. Lysates were layered on 15% sucrose cushion buffer [same composition as lysis buffer but with 15% (w/v) sucrose] and subjected to ultracentrifugation at 55 000 rpm for 50 min at 4 °C in a TLA‐55 rotor (Beckman Coulter, Brea, CA, USA) to separate supernatant and pellet fractions. For analysis of whole‐cell extracts (WCE), cells were harvested and treated with 10% trichloroacetic acid (TCA), and the pellets were resuspended in SDS sample buffer, followed by disruption with glass beads.

Prepared samples (whole‐cell extracts, ribosome pellets, or its supernatant) were separated by SDS–PAGE and transferred to PVDF membranes (Merck Millipore, Darmstadt, Germany). Western blotting was performed using standard procedures with the following primary antibodies: anti‐RPS31 C‐term (1:5000; custom monoclonal against “YKVDAEGKVTKLRR” in the eS31 C‐terminal domain, Sigma‐Aldrich), anti‐PGK1 (1:10000; Invitrogen, Carlsbad, CA, USA, Cat# 459250), anti‐FLAG M2 (1:10000; Sigma‐Aldrich, St Louis Mo, USA, Cat# F3165‐.2MG). Signals were detected with ImmunoStar LD (FUJIFILM Wako, Japan).

### Dual‐luciferase assay

CGA translation bypass and premature termination codon (PTC) readthrough were assayed using genome‐integrated reporters: Rluc‐blank/CGA_12_‐Luc2 (strains Y261 and Y262) and Rluc‐UGG/UGA‐Luc2 (strains S13‐G07 and S13‐G08) (Table S1). For each eS31 variant, the expression plasmid (p414GPD‐eS31 variant) was introduced into both the reporter strain and its paired control strain. Rluc and Luc2 activities were measured in both transformants, and the translation‐through or readthrough efficiency was calculated as the ratio of Luc2 to Rluc in the reporter strain normalized against that of the control strain and expressed as a percentage. In the *UBI3* knockout background, cells were first transformed with p414GPD‐eS31, and the covering plasmid p416GPD‐eS31 was subsequently replaced by plasmid shuffling. Decoding accuracy was assayed using a Rluc–Luc2 WT/H245R misincorporation reporter in p413TEF plasmid. The BY4727ΔeS31 strain (Table S1) was transformed with either WT or H245R reporter plasmid, which served as control and reporter, respectively. Each transformant was then supplied with a second plasmid (p414GPD‐eS31 variants), and the covering p416GPD‐eS31 was replaced by plasmid shuffling. Rluc and Luc2 activities were measured in parallel, and the relative misincorporation rate was determined by comparing the normalized reporter/control ratios.

For paromomycin treatment assays, wild‐type BY4727 cells (no plasmids) were grown overnight, diluted to a balanced OD_600_, and cultured for 6 h in liquid medium with or without 200 μg·mL^−1^ paromomycin prior to lysis and measurement.

For all assays, at least three independent biological replicates were performed. Cells were grown in selective medium, normalized to identical OD_600_ and harvested in log phase. Sample preparation and luciferase activity measurements were performed using the Dual‐Luciferase Reporter Assay System (Promega, Madison, WI, USA) on a SpectraMax iD5 microplate reader (Molecular Devices, San Jose, CA, USA) according to the manufacturer's instructions.

For data presentation, bar graphs show mean ± SD, with individual biological replicates (*n* = 3–5) shown as dots. Statistical significance was assessed by one‐way ANOVA with Dunnett's post‐test (eS31 WT or empty vector as control for eS31 variant comparisons) or paired two‐tailed *t*‐test (for paromomycin effects). Significant differences are denoted as asterisks above the bars (*P < 0.05, **P < 0.01, ***P < 0.001).

### Software and tools for analysis

Molecular graphics and analyses in Figs 1A,B, 2C, 11A, 12, 13 in this paper were performed using the PyMOL Molecular Graphics System, Version 3.1.10 (Schrödinger, LLC). Protein sequences were retrieved from the NCBI Protein or UniProt databases, and accession numbers listed in the corresponding figure legends. Multiple sequence alignment was performed using the MUSCLE algorithm implemented in MEGA 12, and the alignment was visualized using Jalview [62].

## Conflicts of interest

None declared.

## Author contributions

KI, KE supervised projects. QG, HO planned experiments, performed experiments, and analyzed data; KI and QG wrote the paper. KE reviewed the paper.

## Supporting information


**Fig. S1.** Original photo data for yeast growth assays.
**Fig. S2.** Essentiality of eS31 in the S288C‐derived BY4727 genetic background.
**Table S1.** Yeast strains used in this study.
**Table S2.** Plasmids used in this study.

## Data Availability

Genetic materials in this paper are available from the corresponding author [itokoichi@k.u-tokyo.ac.jp] upon reasonable request.
