# Supplementary material for: Genetic dissection reveals distinct contributions of the eS31 N‐terminal domain to translational accuracy in Saccharomyces cerevisiae
Source: FEBS J. 2026 Mar 24;293(20):6076–99. doi: 10.1111/febs.70510 (PMC13669655; doi:10.1111/febs.70510)
Supplement: Supplementary file 1 [file FEBS-293-6076-s001.pdf]

Supporting information

Genetic dissection reveals distinct contributions of the eS31 N-terminal domain to translational accuracy in *S. cerevisiae*

Qingxuan Gao, Hiroshi Otsuka, Kei Endo, Koichi Ito

Department of Computational Biology and Medical Sciences, Graduate School of Frontier Sciences, The University of Tokyo, Kashiwa-city, Chiba 277-8562, Japan

Figure S1. **Original photo data for yeast growth assays.**
Figure S2. **Essentiality of eS31 in the S288C-derived BY4727 genetic background.**
Table S1 **Yeast strains used in this study**
Table S2 **Plasmids used in this study**

Fig S1 (1/3)

Fig. 1D and Fig. 9A

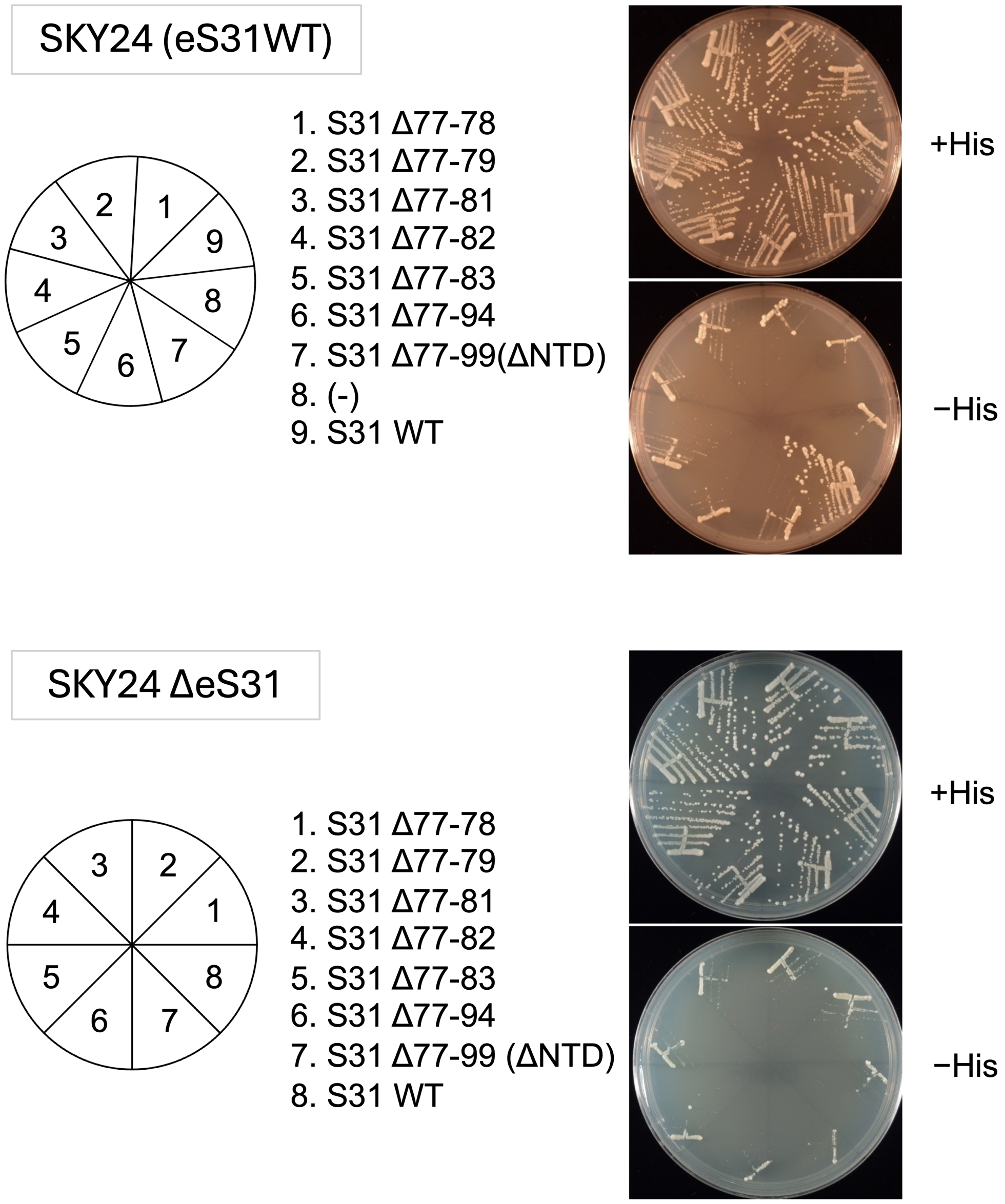

Figure S1. Original photo data for yeast growth assays.

This figure displays the original, uncropped images of the agar plates used for the spot assays shown in the main figures. Each panel corresponds to a specific main figure panel as indicated as in each panel.

Fig. 2B

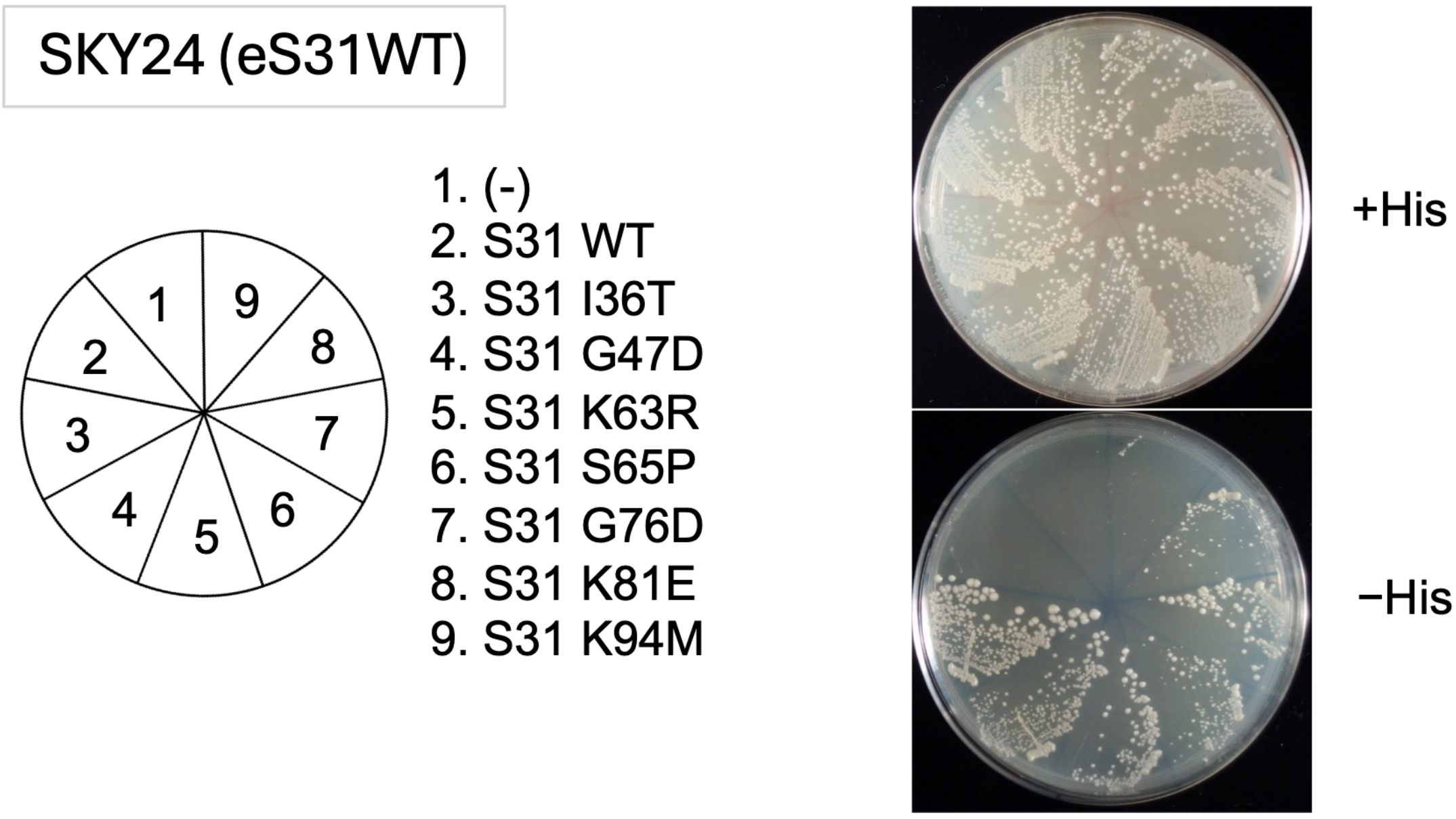
SKY24 (eS31WT)
1. (-)
2. S31 WT
3. S31 I36T
4. S31 G47D
5. S31 K63R
6. S31 S65P
7. S31 G76D
8. S31 K81E
9. S31 K94M
+His
−His

Fig. 2D

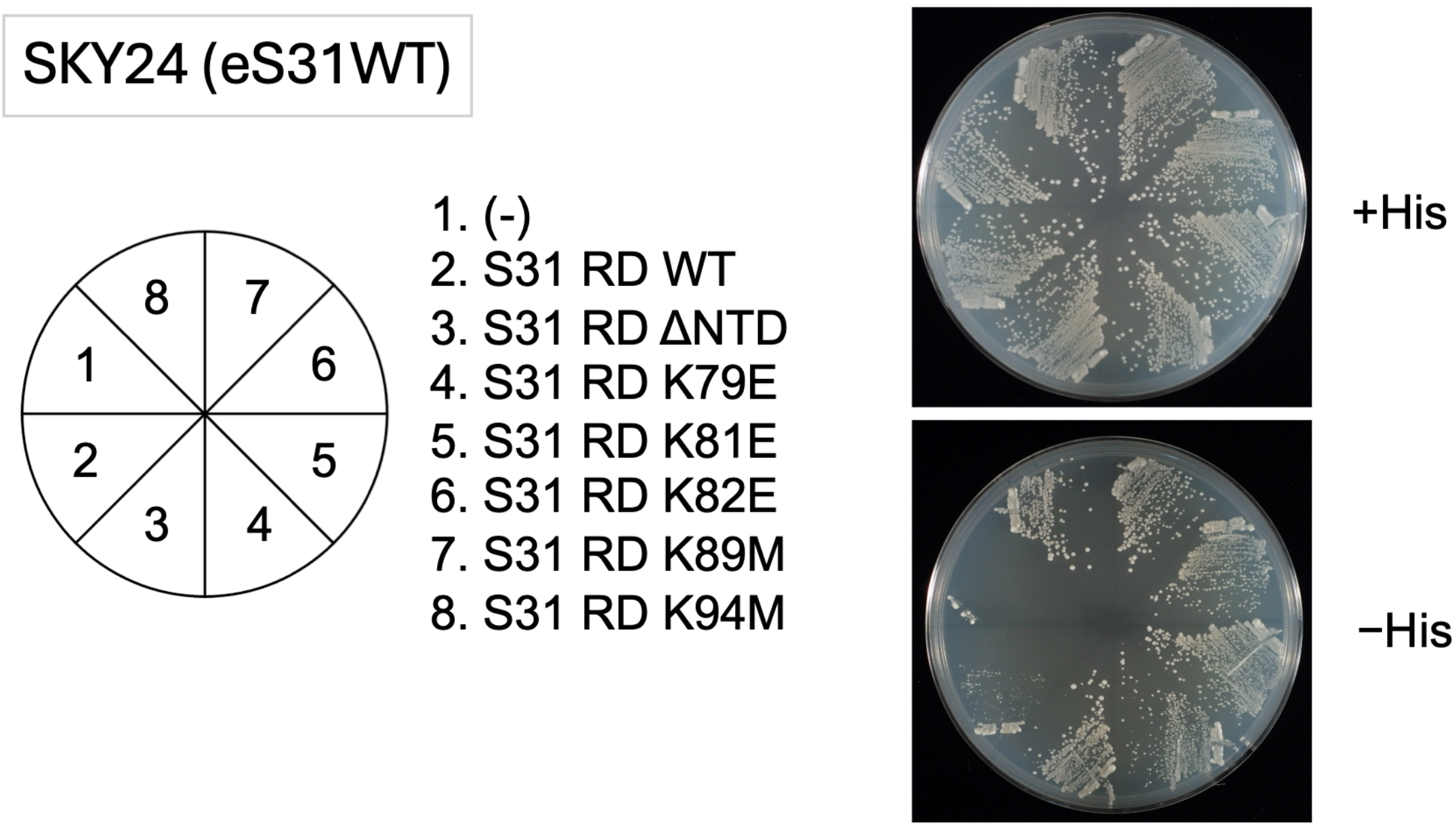
SKY24 (eS31WT)
1. (-)
2. S31 RD WT
3. S31 RD ΔNTD
4. S31 RD K79E
5. S31 RD K81E
6. S31 RD K82E
7. S31 RD K89M
8. S31 RD K94M
+His
−His

Fig S1 (3/3)

Fig. 5B

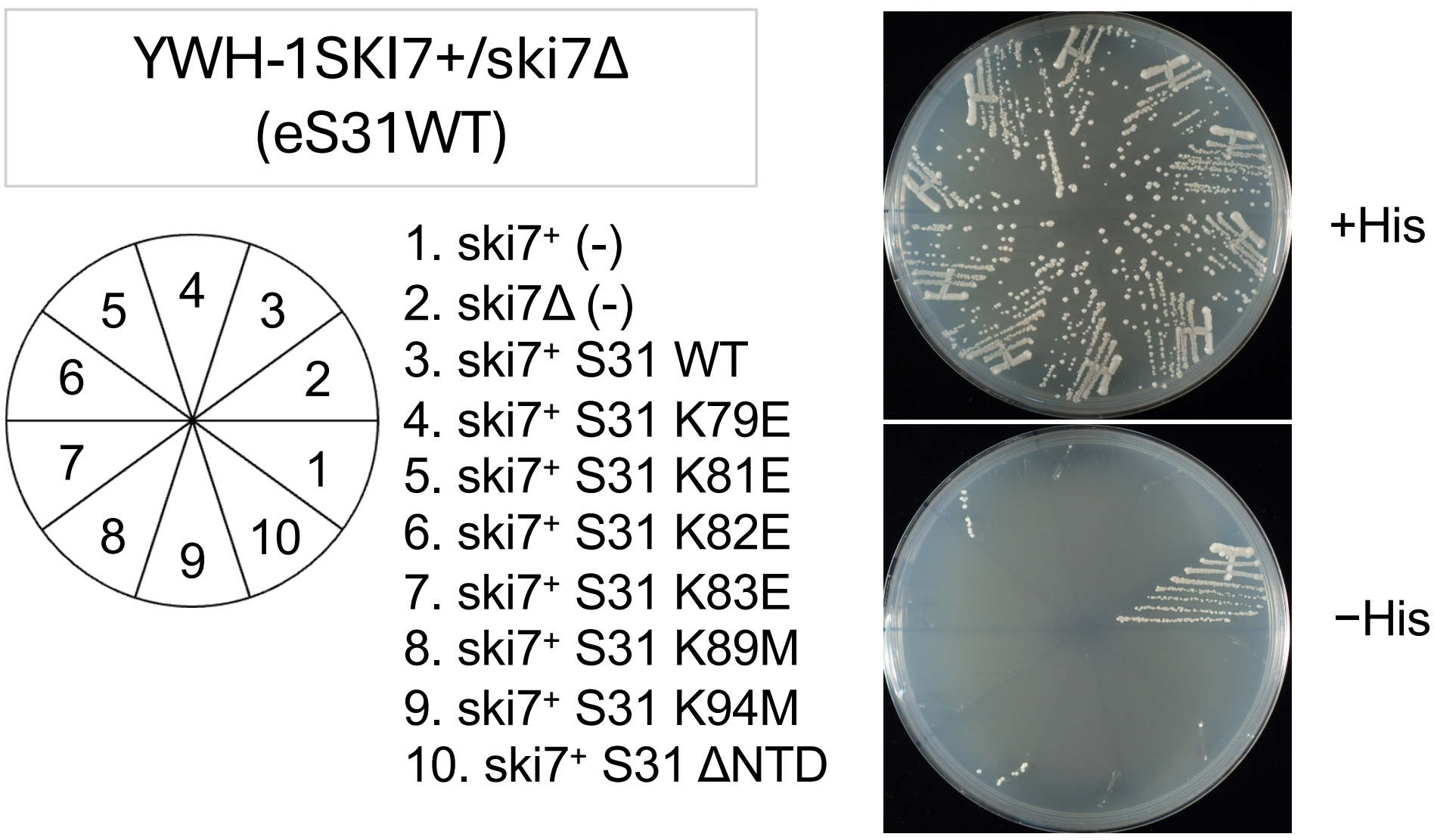

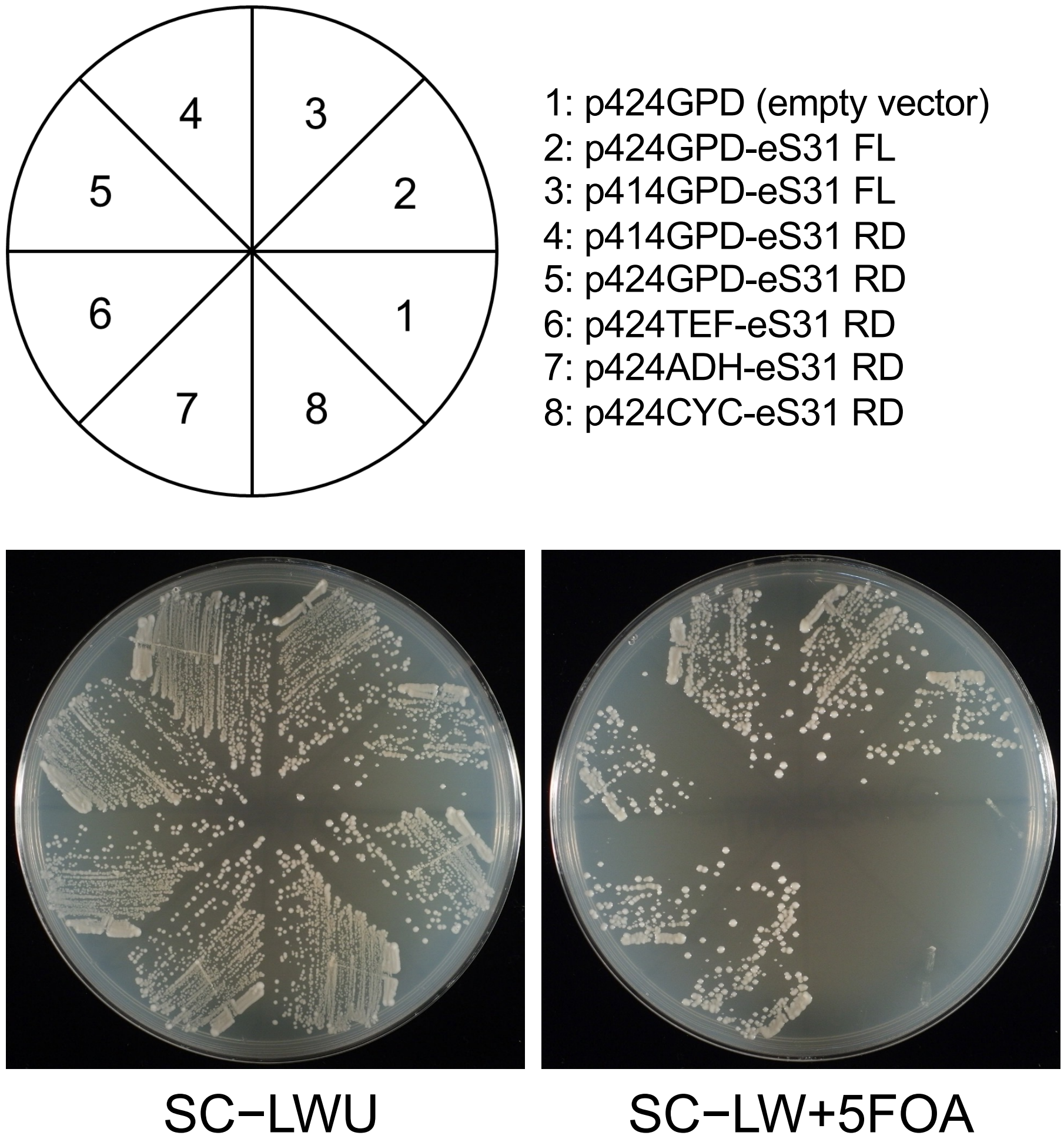

Figure S2. Essentiality of eS31 in the S288C-derived BY4727 genetic background. Plasmid shuffling assay confirms that *RPS31* (*UBI3*) is essential for viability in the BY4727 strain. Strains carrying the *URA3*-marked covering plasmid (SKY24 ΔeS31 p416GPD-*UBI3*) were transformed with the indicated plasmids (1–8) and selected on SC-Leu-Trp-Ura (SC-LWU) medium (left). Cells were then streaked onto SC-Leu-Trp medium containing 5-fluoroorotic acid (5-FOA) to select for the loss of the covering plasmid (right). Only cells expressing functional eS31 variants (Full-length [FL] or Ribosomal Domain [RD]) from the indicated promoters were able to grow on 5-FOA, whereas cells with the empty vector (1) failed to grow, demonstrating the inviability of the *ubi3Δ* background. Notably, the failure of the weakest CYC promoter (8) to support growth, in contrast to the stronger GPD (5), TEF (6), and ADH (7) promoters, is consistent with the requirement for a minimum threshold of eS31 expression to maintain cellular viability. Images shown are representative of results obtained from at least three independent transformants tested in parallel, all of which exhibited reproducible growth phenotypes.

Table. S1 Yeast strains used in this study

BY4727 [a]	MAT alpha, *his3Δ200 leu2Δ0 lys2Δ0 met15Δ0 trp1Δ63 ura3Δ0*	[76]
BY4727ΔeS31	BY4727 *UBI3::KL-LEU2* + p416GPD-eS31	This study
BY4727Δasc1Δhel2	BY4727 *ASC1::kanMX HEL2::hphMX*	[43]
SKY24	BY4727 *HO::TEFp-Rluc-CGAx12-HIS3-CYCt-kanMX*	[42]
	(CGA_{12}–*HIS3* reporter strain)	
SKY24ΔeS31	SKY24 *UBI3::Kl-LEU2* + p416GPD-eS31	This study
SKY24Δdph2	SKY24 *DPH2::KL-URA3*	This study
SKY24Δdom34	SKY24 *DOM34::delta*	This study
SKY24Δhbs1	SKY24 *HBS1::delta*	This study
Y218	BY4727 *RPL25*-FLAG-His6	[43]
Y218ΔeS31	Y218 *UBI3::LEU2* + p416GPD-eS31	This study
S13-G07	BY4727 can1-100(UAA) ade2-1(UAA) *HO::GPD-Rluc-[UGA]-Luc2-CYCt*	Laboratory stock
S13-G08	BY4727 can1-100(UAA) ade2-1(UAA) *HO::GPD-Rluc-[UGG]-Luc2-CYCt*	Laboratory stock
Y261	BY4727 *HO::TEFp-Rluc-blank-luc2-CYCt- kanMX*	[40]
Y261ΔeS31	Y261 *UBI3::LEU2* + p416GPD-eS31	This study
Y262	BY4727 *HO::TEFp-Rluc-CGAx12-luc2-CYCt- kanMX*	[40]
Y262ΔeS31	Y262 *UBI3::LEU2* + p416GPD-eS31	This study
YWH-1SKI7+	BY4727 *HO::TEFp-HIS3ns-HIS3t-kanMX*	[48]
YWH-1ski7Δ	BY4727 *HO::TEFp-HIS3ns-HIS3t-kanMX SKI7::hphMX*	[48]

[a]: BY4727 is a derivative of the S288C genetic background

Table. S2 Plasmids used in this study

p413TEF	TEF promoter CEN6/ARSH4 HIS3 marker	[71]
p414GPD	GPD promoter CEN6/ARSH4 TRP1 marker	[71]
p415ADH	ADH promoter CEN6/ARSH4 LEU2 marker	[71]
p416GPD	GPD promoter CEN6/ARSH4 URA3 marker	[71]
p413TEF-Rluc-Luc2	Misincorporation reporter (control) in p413TEF	This study
p413TEF-Rluc-Luc2 H245R	Misincorporation reporter in p413TEF	This study
p414GPD-eS31 (RD)	eS31 (RD) WT/mutants in p414GPD	This study
p416GPD-eS31 (RD)	eS31 (RD) WT/mutants in p416GPD	This study
p416GPD-Hel2	Hel2 in p416GPD	This study
p416GPD-Dom34	Dom34 in p416GPD	This study
p416GPD-Hbs1	Hbs1 in p416GPD	This study
p416GPD-Dph2	Dph2 in p416GPD	This study
p415ADH-Hel2-TRP1C45	Hel2–TRP1C45 fusion in p415ADH	[43]
p413TEF-Asc1-TRP1N44	Asc1–TRP1N44 fusion in p413TEF	[43]
